# Comprehensive metagenomic and lipidomic analysis showed that baicalin could improve depressive behaviour in atherosclerotic mice by inhibiting nerve cell ferroptosis

**DOI:** 10.3389/fimmu.2025.1599570

**Published:** 2025-09-05

**Authors:** Peng Ren, Yulong Zhao, Xue Li, Jing Xie, Xingxing Liao, Qiang Luo, Xu Liu, Jiameng Li, Yuzhen Fan, Xinyi Cheng, Xinyao Fu, Junjie Zhou, Xiaoyun Wu

**Affiliations:** ^1^ School of Rehabilitation Medicine, Gannan Medical University, Ganzhou, Jiangxi, China; ^2^ School of Rehabilitation, Capital Medical University, Beijing, China; ^3^ Beijing Bo’ai Hospital, China Rehabilitation Research Center, Beijing, China; ^4^ Key Laboratory of Prevention and Treatment of Cardiovascular and Cerebrovascular Diseases of Ministry of Education, Gannan Medical University, Ganzhou, China; ^5^ Ganzhou Key Laboratory of Rehabilitation Medicine, Gannan Medical University, Ganzhou, Jiangxi, China; ^6^ School of Basic Medicine, Gannan Medical University, Ganzhou, Jiangxi, China

**Keywords:** atherosclerosis, baicalin, gut microbiota, lipid metabolism, ferroptosis, depression

## Abstract

**Background:**

Atherosclerosis (AS) concomitant depression is a serious clinical problem with unclear mechanisms of co-morbidity. Baicalin (BA) can resist atherosclerosis and depression by regulating intestinal flora and host lipid metabolism. Therefore, based on intestinal microorganisms and lipid metabolism, this study explored the mechanism of baicalin against AS concomitant depression.

**Methods:**

16 C57BL/6 mice were fed with normal diet as blank control group. 48 ApoE^-/-^mice were randomly divided into 3 groups (model group and BAL, BAH two treatment groups). The mouse model of atherosclerosis concomitant depression was established by high-fat feeding combined with restraint stimulation for 16 weeks. Behavioural experiments and biochemical indexes were used to detect the antidepressant effect and anti-atherosclerosis effect of baicalin. Metagenomic sequencing technology combined with metabolomics analysis was used to detect the effects of BA on intestinal microflora structure and brain lipids in AS co-depressed mice. Erastin was used to induce HT-22 hippocampal neurons to construct a model of ferroptosis. The inhibition of baicalin on ferrotosis was verified by detecting the cell viability, ROS production, and expression levels of glutathione, SLC7A11, GPX4 and ACSL4 in each group.

**Results:**

Baicalin could effectively improve the indexes of AS co-depressed mice, and the results of metagenomics and lipidomics showed that there were disorders of intestinal flora represented by Helicobacter_typhlonius and Escherichia_coli and disorders of lipid metabolism represented by PE in the AS co-depressed model mice. The correlation analysis showed that the lipid metabolism disorders in the model mice were closely related to the intestinal flora disorders, and baicalin intervention could effectively improve the intestinal flora and lipid metabolism disorders in the AS co-depressed mice. Metabolic pathway enrichment analysis showed that differential lipid PEs were significantly enriched in the iron death pathway, and our further *in vitro* cellular experiments showed that baicalin could inhibit Erastin-induced Ferroptosis in the hippocampal neuronal cell line HT-22 by promoting the expression of SLC7A11, GSH, and GPX4, inhibiting the expression of ACSL4, and decreasing the cellular ROS.

**Conclusion:**

Baicalin improves intestinal microbiota and brain lipid metabolism and inhibits ferroptosis of nerve cells, which possesses the application value of anti-atherosclerotic concomitant depression.

## Highlights

- Baicalin improved the indexes of AS co-depression model mice.
- Baicalin improved gut microbiota disturbance in AS co-depression mice.
- Baicalin ameliorates lipid metabolism disorders in AS co-depression mice with differential lipids enrichment in the ferroptosis pathway.
- Baicalin inhibited ferroptosis in neurons.

## 1 Introduction

Recently, AS co-depression disease has drawn more attention. One of the conditions with the greatest morbidity and death rates in the world, atherosclerosis is the pathologic foundation of cardiovascular disorders, such as peripheral vascular disease, cerebral infarction and coronary heart disease (1). Depression is a prevalent, long-lasting, and severe mental disease that is marked by a recurring sense of sadness, irregular sleep patterns, loss of appetite or weight, social anxiety, etc. (2). According to epidemiologic studies, numerous vascular risk factors, like obesity, hypertension, and hyperlipidemia, contribute to the higher prevalence of depression in individuals with AS (3, 4). Depression in individuals with AS can easily result in cardiovascular and cerebrovascular emergencies, raise patient mortality rates, decrease medication efficacy, and negatively impact patients’ quality of life and prognosis. All of these factors significantly worsen the financial burden of medical care on families and society (5). Given the dearth of specific pathogenesis of AS co-depression, and the clinical therapy available for AS co-depression is facing great difficulties, it is imperative to clarify the distinct pathophysiology of AS co-depression and to develop more efficacious preventive and therapeutic approaches (6).

The potential co-pathogenesis between AS and depression is quite complex, and multiple mechanisms are currently thought to be involved, such as gut microbiota disorder, lipid metabolism disorders, inflammation, the hypothalamic–pituitary–adrenal (HPA) axis hyperactivity, and autonomic dysfunction (7). According to recent research, the gut microbiome, which is able to manipulate the metabolism of the host, plays a significant part in the pathogenesis of atherosclerosis (8–10) and may also be a principal factor in the development of depression (11–13) (Barrington & Lusis, 2017; P. B. Chen et al., 2020; Komaroff, 2018). By assisting in the control of host lipid metabolism, gut microbiota and their byproducts can have an impact on metabolic illnesses (14, 15) (Li et al., 2024). Lipids are both a pivotal factor in atherosclerotic lesions (16) and an important component of neuronal cell membranes within the brain, vital to the survival and signalling of neurones (17, 18), and several investigations have shown that depression is associated with aberrant lipid metabolism (19, 20). Consequently, we suggest that a significant contributing component to the pathophysiology of AS co-depression may be lipid metabolism problems based on altered gut microbiota, but more experimental data are demanded to elucidate the specific molecular mechanisms.

BA, a kind of flavonoid component that was identified from Scutellaria baicalensis Georgi’s root, has both anti-AS activities, such as hypolipidemic and anti-inflammatory, and antidepressant effects, such as neuroprotection, and improved cognition and memory. Research has indicated that by altering the structure and abundance of gut microbiota, BA also impacts glucose homeostasis, lipid and energy metabolism, depression, and cardiovascular disease (21–23), making BA a potentially effective natural compound for preventing and ameliorating co-depression in AS. This research aims to examine the impact of BA as a co-depressant against AS from the perspective of gut microbiota, providing more experimental data support for the prevention and treatment of atherosclerosis co-depression diseases.

In this study, BA was used to treat the animal model of AS concomitant depression ApoE^-/-^ mice obtained by high-fat combined bind, and the results showed that BA was effective in improving the depression-like behaviours and various indexes of AS in the AS co-depression model mice. Subsequently, upon taking the faeces of AS co-depression mice for metagenomics sequencing, it was observed that the gut microbiota of the high-fat combined bind group (HFB) was markedly and significantly altered in comparison to the normal control group (NC), with BA improving the corresponding gut microbiota disorders; Mice hippocampus and prefrontal cortex tissues were taken for lipidomics, which indicated that some lipids were significantly altered in the HFB compared with that of the NC group, for example, PE, and BA improved the related lipid profile, while BA improved the related lipid disorders, and the KEGG database-based metabolic pathway enrichment analysis of the differential metabolites revealed a strong enrichment in the ferroptosis pathway. Combined metagenomic and lipidomic analyses revealed that significantly altered microorganisms such as *Helicobacter typhlonius* and *Escherichia coli* are closely associated with lipid PE in the hippocampus and prefrontal cortex, as well as the most sensitive and important substrate for lipid peroxidation in the signalling pathway of ferroptosis, based on our literature assessment, PEs are critical for the formation and functionality of neuronal cell membranes (24). We then further demonstrated by *in vitro* cellular experiments that BA effectively inhibited erastin-induced ferroptosis in the hippocampal neuronal cell line HT-22 and exerted neuroprotective effects. To sum up, our study demonstrated that BA exerted its anti-AS co-depression effects by improving the intestinal flora, regulating lipid metabolism, and inhibiting ferroptosis.

## 2 Materials and methods

### 2.1 BA preparation

BA was acquired from Pester Biotechnology Co. BA powder was dissolved in saline before use. In the low-dose group, the gavage dosage of BA was 50 mg/kg, whereas in the high-dose group, it was 100 mg/kg.

### 2.2 Animals

16 male C57BL/6J mice and 48 ApoE^-/-^mice (8 weeks old, 20 ± 2 g, SPF grade) were purchased from Jiangsu Huachuang Xinnuo Pharmaceutical Science and Technology Co Ltd (No. 202233341). The habitat in which the animals were kept with humidity of 55 ± 5%, temperature of 22 ± 1 °C, alternating light/darkness for 12 h per day, and unrestricted food and water supply. All animals are kept and operated in strict accordance with national regulations on experimental animal management and the guidelines of the Animal Husbandry Ethics Committee of the Experimental Animal Centre of Gannan Medical University, and reported to the Experimental Animal Ethics Committee of Gannan Medical University for the record(NO.2025439).

### 2.3 Model preparation and treatment

Based on the principle of statistically randomised grouping, 48 ApoE^-/-^ mice were randomly divided into three groups: HFB group, baicalin low-dose (BAL; 50 mg/kg/d) group, baicalin high-dose (BAH; 100 mg/kg/d) group. The mice were fed a high-fat diet (0.15% cholesterol, 21% fat) on a daily basis. The mice in the HFB, BAL and BAH group were bound to a 50ml cryopreservation tube for one hour every day. The mice in the HFB group were gavaged with saline (10 ml/kg) once per day. The mice in BAL and BAH group received baicalin intervention once a day. The modelling was performed concurrently with the BA intervention until the end of the experiment. The normal control group (NC group) comprised 16 C57BL/6J mice, which were provided with a normal diet and gavaged with saline (10 ml/kg) on a daily basis until the conclusion of the experiment. The intervention dose of baicalin was adjusted on a weekly basis, based on the weight of the mice.

### 2.4 Behavioural experimental analysis

#### 2.4.1 Sucrose preference test

Two times was a sucrose preference test started: initially in the 0th week and subsequently in the 16th week. The experiment was conducted over a period of three days. During the initial two days, the subjects were allowed to acclimatise. During this period, two bottles of 1% sucrose solution were provided to each cage. After 24 hours, the sucrose solution was replaced with two bottles of drinking water. Following the acclimatisation training, the sucrose preference test was conducted in accordance with the standard protocol, whereby equal volumes of drinking water and 1% sucrose water were provided in each cage. To prevent any potential bias due to positional preference on the accuracy of the experimental results, the positions of the two water bottles were exchanged once after 12 hours. The consumption of drinking water and sucrose water by each mouse was then measured and recorded 24 hours later. The sucrose preference rate(%) = sucrose water consumption/total fluid consumption × 100%. Mice were kept in their original environment after the experiment.

#### 2.4.2 Open field test

The experimental apparatus, measuring 25cm x 25cm x 35cm, was situated within an open-mouthed box. In a dark and quiet environment, at the bottom of the box, each mouse was positioned in the middle of the grid, and the activity of the mice was recorded via an infrared camera for a period of five minutes. Subsequently, the total distance and total time spent in the centre area of each mouse were subjected to statistical analysis using an automated mouse tracking system (Ethovision XT 11.5). At the conclusion of each mouse trial, the olfactory stimulus associated with the mouse within the testing apparatus was eliminated to prevent any potential interference with the subsequent mouse experiment.

#### 2.4.3 Tail suspension experiment

At the finish of week 15, the mice were placed in a 20 cm × 20 cm × 40 cm black plastic observation box, situated 35 cm above the ground, and observed for a period of six minutes. This was achieved by the application of medical tape to a 1 cm segment of the tail tip of each mouse. It’s crucial to remember that the experimental mice were completely isolated from one another during the test, in order to avoid any potential visual and auditory interferences. At the conclusion of the trial, the odour of the mice in the observation box was eliminated. The immobility time of the mice in the suspension tail box was recorded using an automated tracking system for mice (Ethovision XT 11.5).

### 2.5 Analysing the indications of atherosclerosis

#### 2.5.1 Blood lipid detection

Mice were put in deep anaesthesia with a mixture of 4–5% isoflurane and O2. Arterial blood was collected into 1.5 ml centrifuge tubes. After centrifugation, the supernatant was taken. Blood samples from each group of mice were tested using a high-density lipoprotein cholesterol (HDL-C) test kit (Item No.: A112-1-1), a low-density lipoprotein cholesterol (LDL-C) test kit (Item No.: A113-1-1), a total cholesterol (T-CHO) test kit (Item No.: A111-1-1) and a triglyceride (TG) test kit (Item No.: A110-1-1). All the animal experiments included in this study were carried out according to ARRIVE guidelines.

#### 2.5.2 Oil red O staining was used to identify the development of plaque in the mouse thoracic aorta

The adherent tissue of the aortic epithelium was removed microscopically with instruments, and the aortic vessels were cut longitudinally with surgical scissors along the side of the lesser curvature of the aortic arch site. Thereafter, the aorta was fixed in a 4% paraformaldehyde solution for 24 hours. The fixed specimens were removed and washed with deionised water for 15 min to remove the residual paraformaldehyde from the vessels. The aorta was dyed for 2 hours at room temperature after being fully immersed in oil red O working solution. The aortic vessels were removed from the working solution, differentiated by 70% ethanol, and the images were captured with a high-definition digital camera after the residual ethanol solution was removed by deionised water.

#### 2.5.3 HE staining is used to identify histological alterations in coronary arteries

HE staining was used to observe the morphology of coronary arteries. The fixed mice aorta tissues were removed from 4% paraformaldehyde solution, and the coronal surface was continuously sliced with conventional paraffin embedding. First, the sheets were dried in a 60°C dryer for 2 h, followed by paraffin dewaxing and hydration, and soaked in xylene twice for 10 min each time. The slices were then immersed in ethanol solution of different concentrations (100%, 95%, 85%, 75%) for 5 min, then gave it a five-minute rinse with distilled water. After that, distilled water was used to wash the floating colour, differentiate the differentiation liquid for 3 minutes, and wash the distilled water twice for a total of two minutes each after the haematoxylin was stained for 5 minutes. The specimens were soaked in distilled water for three to 5 minutes following eosin staining. After being dried for 5 minutes every layer using a gradient alcohol (95–100%), the layers were transparent for one minute each time after being transparently sealed with neutral gum, examined under an optical microscope, and captured on camera.

### 2.6 Microbial community analysis using metagenomic sequencing

On the last day of the trial, mice’s excrement from each of the six groups (n = 6) was collected. For the purpose of extracting DNA, four to five faeces (about 250 mg) from each mouse were gathered in 1.5 ml enzyme-free centrifuge tubes that had been marked beforehand, snap-frozen in liquid nitrogen, and then quickly kept in a refrigerator at 80 °C. Mag Pure Stool DNA KF Kit B (MAGEN, Guangzhou, China) was used to extract DNA from samples in accordance with the handbook’s instructions. Using a high-speed grinder (Shanghai Jingxin Science and Technology, China), 100–200 mg of the material should be ground and put into a centrifuge tube with grinding beads. Fill the buffer ATL/PVP-10 with 1 mL. At 65 °C, incubate the lysis for 20 minutes. After that, it was placed in an Eppendorf centrifuge (Germany) and centrifuged for five minutes at 14,000 g. The resulting fluid was then transferred to a brand-new centrifuge tube and filled with 0.6 millilitres of Buffer PCI. It was then vortexed and mixed for 15 seconds, then centrifuged for 10 minutes at 18213 g. At last, the mixture was moved to a deep well plate that contained a mixture of 600μL Buffer Magnetic Bead Binding Solution + 20μL Proteinase K + 5μL RNase A, 700μL Wash 1, 700μL Wash 2, 700μL Wash 3, and 100μL Elution Buffer that had been spiked with magnetic bead binding solution. Start Kingfisher (Kingfisher, Thermo Fisher, USA), select the corresponding program, place each deep-well plate in the corresponding position of the instrument, and run the program. Once the procedure is complete, transfer the DNA solution from the deep-well plate with the elution buffer to a 1.5 mL centrifuge tube for storage.

A certain quantity of genomic DNA was extracted and broken apart. Magnetic bead fragment selection was used to samples that had interruptions. To add an A base to the 3’ end and repair DNA ends, set up a reaction system and reaction program; to link the junction to the DNA, create a junction joining reaction system and program. To amplify the products, set up the PCR reaction apparatus and reaction software. Choose the right assay based on the product specifications for the library’s quality control.

Single-stranded DNA library molecules are obtained by subjecting the libraries that pass quality control to denaturation processes. The uncyclised linear DNA molecules are then digested, and a cyclisation reaction system yields the single-stranded cyclic product. Through the replication processes of phi29 and roll-over, single-stranded circular DNA molecules combine to produce a DNA nanoball (DNB) that contains multiple copies. Through the replication processes of phi29 and roll-over, single-stranded circular DNA molecules combine to produce a DNA nanoball (DNB) that contains multiple copies. Using high-density DNA nano-chip technology, the generated DNBs were spiked into the chip’s mesh pores. They were then subjected to PE100/PE150 sequencing on the DNBSEQ-G400/T7/T10 sequencing platform by combining probe-anchored polymerisation (CPAs).

### 2.7 Lipid differential metabolites were identified in the hippocampus and prefrontal cortical tissues of mice

The Waters UPLCl-Class Plus (Waters, USA) tandom Q Exactive high resolution mass spectrometer (Thermo Fisher Scientific, USA) was utilised in this experiment to separate and identify metabolites.

Chromatographic conditions: Chromatographic separation was performed on CSH C18column (1.7 um 2.1*100 mm, Waters, USA). At positive ion mode with mobile phase A consisting 60%acetonitrile in water + 10mM ammonium formate + 0.1% formic acid and mobile phase B consisting 90% isopropanol + 10% acetonitrile + 10mM ammonium formate + 0.1% formic acid. At positive ion mode, with mobile phase A consisting 60% acetonitrile in water + 10mM ammonium formate and mobile phase B consisting 90% isopropanol+ 10% acetonitrile + 10 mM ammonium formate. The column temperature was maintained at 55°C. The gradient conditions were as follows: 40% ~43% B over 0~2min,43%~50% Bover 2~2.1min,50%~54% B over 2.1~7 min,54%~70% B over 7~7.1min,70%~99% B over 7.1~13min,99%~40% B over 13~13.1 min, held constant at 99%~40%B over 13.1~15 min and washed with 40% B over 13.1-15min. The flow rate was 0.4 mL/min and the injection volume was 5μL.

Mass spectrometry conditions: Using Q Exactive (Thermo Fisher Scientific, USA) perform primary and secondary mass spectrometry data acquisition. The full scan range was 70–1050 m/z with a resolution of 70000, and the automatic gain control (AGC) target for MS acquisitions was set to 3e6 with a maximum ion injection time of 100ms, Top 3 precursors were selected for subsequent MSMS fragmentation with a maximum ion injection time of 50ms and resolution of 17500, the AGC was le5, the stepped normalised collision energy was set to 15, 30 and 45 eV. ESI parameters were setting as: Sheath gas flow rate was 40, Aux gas flow rate was 10, positive-ion mode Spray voltage(|KV|)was 3.80, negative-ion mode Spray voltage(|KV|) was 3.20,Capillary temperature was 320 °C, Aux gas heater temperature was 350 °C. See **Supplementary Methods** for methodological details of lipidomics.

### 2.8 Microbial taxa associated with lipid differential metabolites: an association analysis

Based on the rank value of each variable (rather than the raw data), Spearman’s correlation coefficient determines the monotonic relation between two continuous or sequential variables. Spearman’s coefficient is applicable to both continuous and discretely ordered variables.

### 2.9 Cell culture

The HT-22 cell line (Item No. AW-CNM116) was acquired from Abiowell Biotechnology Co. Ltd. and developed at 37 °C in 5% CO2 DMEM medium with 10% FBS+1% and double antibody, at saturated humidity in an incubator. The logarithmically grown cells were taken and spread inside a 6-well plate, and after the cells were attached to the wall, they were processed in the following grouping: (1) Blank control group: cells were cultured normally for 24 h;(2) Model group: add erastin (10μM) to establish ferroptosis model treated for 24 h;(3) BAL (3μM) treatment group: pre-treated with BA (3μM) for 1 h and then co-treated with erastin (10μM) for 24 h;(4) BAM (9μM) treatment group: pre-treated with BA (9μM) for 1h and then co-treated with erastin (10μM) for 24h;(5) BAH (27μM) treatment group: pre-treated with BA (27μM) for 1h and then co-treated with erastin (10μM) for 24h.

### 2.10 Cell viability assay

A density of 5×10^3^ cells/well was achieved by injecting 100 µL of logarithmically grown, digested, and counted cells onto 96-well plates. Each group was provided with three replicated holes. After the culture is attached to the wall and processed as above for the appropriate time, add 10µL of CCK8 per well as described above, configure the CCK8 solution with complete medium, and remove the drug-containing medium by adding 100 µL of CCK8-containing medium per well. After four hours of incubation at 37 °C and 5% CO2, the absorbance (OD) value at 450 nm was measured using a Huisong enzyme marker.

### 2.11 ROS assay

To get a final concentration of 10 um, dilute DCFH-DA (master mix concentration 10 mM) with serum-free culture medium in accordance with a 1:1000 ratio. After the above-treated cells were centrifuged to get the cell precipitate, 500 millilitres of the configured working solution were added to resuspend the cells. For twenty minutes, the cells were grown in a cell culture incubator at 37 °C. Serum-free cell culture solutions were utilised to wash the cells three times in order to effectively remove DCFH-DA that had not entered the cells. Flow cytometry was employed with the apparatus.

### 2.12 Glutathione assay

Following the manufacturer’s instructions, the Trace Reduced Glutathione (GSH) Assay Kit (Item No.: A006-2-1) from Nanjing Jiancheng Bioengineering Institute was used to detect the glutathione concentration in cell lysates.

### 2.13 Western blot

Protein extraction from HT-22 cells. After a single washing with ice-cooled PBS, 200μL of RIPA lysate was added to the cells. Following the collection, lysing, and centrifuging of the suspension, a centrifuge tube was filled with the supernatant. By following the BCA protein measurement kit’s instructions, the protein content was ascertained. Take 100μL of protein supernatant, add 25μL of 5×loading buffer, and mix well. Cook in boiling water for 5 minutes, then put it into an ice box for speedy cooling. Denatured proteins were electrophoresed using electrophoresis at a constant voltage of 80 V for 100 min. Electrophoresis was applied to distinguish the denatured proteins, with a continuous voltage of 80 V for one hour. When the bromophenol blue electrophoresis hits the bottom of the gel, stop the process. A steady current of 300 mA was used to transfer the membranes; the antibody information provides the transfer times for each indication. After preparing the membranes with 1×PBST and closing them with 5% skim milk powder, primary and secondary antibodies were added and incubated. exposed using a gel imaging device and imaged with an ECL colour development system.

### 2.14 Data statistics

Statistical analyses were performed using SPSS 23.0 (IBM, USA) and R 4.1.2 (R Foundation). Data processing and hypothesis testing followed these procedures: Group comparisons: Continuous variables with normal distribution (assessed by Shapiro-Wilk test) were analysed using one-way ANOVA or two-way ANOVA with Tukey’s *post hoc* test. Non-normally distributed data or ordinal variables were evaluated with non-parametric tests: Kruskal-Wallis test for >2 groups (followed by Dunn’s correction) Mann-Whitney U test for two-group comparisons. Alpha diversity of the species was calculated using the R package, including the Chao1 index, the Shannon index, and the Simpson index. Differences between samples or groups were also measured by calculating the Bray-Curtis distance (25) and JSD distance (Jensen-Shannon divergence) (26), i.e., beta diversity (27), which reflects whether or not the samples (groups) have significant microbial community differences. Abundance data on the composition of microbial communities is usually tested using the non-parametric Wilcoxon/Kruskal-Willas test. The metabolomics R package metaX was used to do statistical analysis on the Lipidomics LC-MS/MS data, which were processed using LipidSearch 4.1 software (28). The student’s t-test was utilised to conduct significance testing for the expression of every metabolite in every comparison group. The R software core was utilised to implement Spearman’s correlation analysis. Analyse the sequencing data from the lipome and microbiota together.

## 3 Results

### 3.1 BA improves depressive-like behaviours in AS co-depression mice

**Figure 1:**
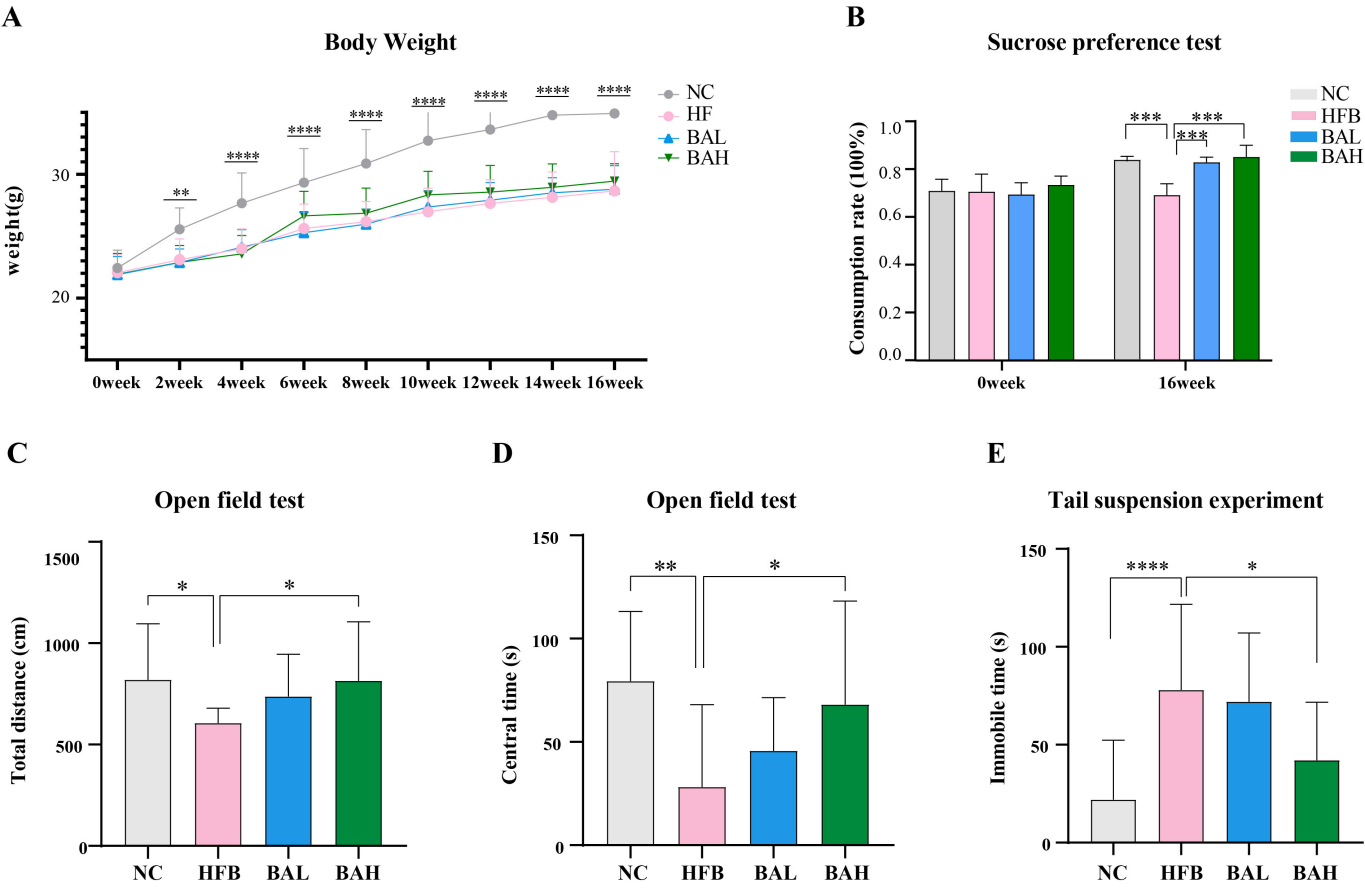
BA improves depression-like behaviours in AS-co-depression mice. **(A)** Body weight change in mice. **(B)** Sucrose preference rate. **(C)** The entire distance of open field test. **(D)** Duration of the open field test in the centre. **(E)** Tail suspension experiment: suspended immobile time. All values are presented as mean ± SEM (N=16). *, P < 0.05; **, P < 0.01; ***, P < 0.005; ****, P < 0.001, vs HFB.

Depression-related indicators in AS co-depression mice were assessed using body weight test, sucrose preference test, Open field test, and hanging tail test. The results of the body weight test showed that the mice in the HFB group had significantly lower body weights compared with the NC group (**Figure 1A**), the sucrose intake of mice in the HFB group was significantly lower than that in the NC group(**Figure 1B**), the total movement distance and the total time in the centre zone of the open field test were significantly lower than those of the NC group (**Figures 1C, D**), and the resting time in the tail-hanging experiments was significantly increased (**Figure 1E**), while the above indicators were improved when obtained after BA intervention, indicating that BA could alleviate depression-like behaviour.

### 3.2 BA improves atherosclerosis-related indices in AS co-depression mice

**Figure 2:**
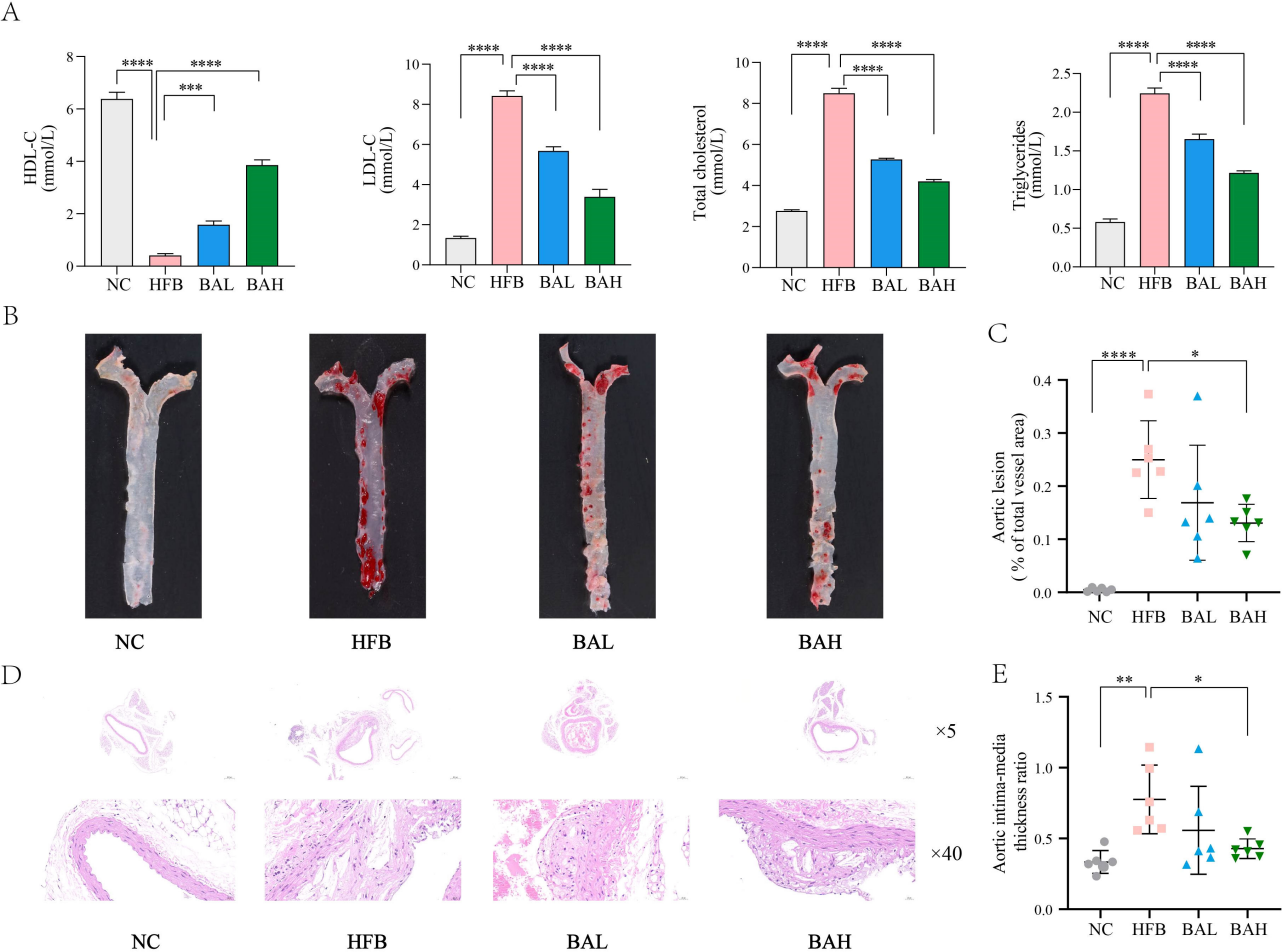
BA inhibits atherosclerotic lesion formation in ApoE-/- mice. **(A)** Mice plasma lipid levels. **(B)** Aorta oil red O staining at 16 weeks in mice. **(C)** Quantitative statistics of plaque area in the mice thoracic aorta. **(D)** HE staining of the coronary surface of aorta (at 16 weeks; ×5, 5-fold; ×40, 40-fold). **(E)** Intima-media thickness ratio. All values are presented as mean ± SEM (N=6). *, P < 0.05; **, P < 0.01; ***, P < 0.005; ****, P < 0.001.

Chronic, progressive atherosclerosis is characterised by abnormalities in lipid metabolism that raise blood cholesterol and cause atheromatous plaque lesions (29). Therefore, atherosclerosis related indices in AS co-depression mice were assessed by measuring plasma levels of LDL-C, HDL-C, TG, and TC, and by pathological staining of the aorta. The results of blood lipid analyses showed that the high-fat diet induced significant elevation of TC, TG, and LDL-c as well as significant reduction of HDL-c in ApoE^-/-^ mice, and the BA intervention was able to significantly ameliorate all the abnormal lipid levels in AS co-depression mice (**Figure 2A**). Regarding the results of aortic oil red O staining, the aortic plaque area was increased in the HFB group of mice, and the BA intervention reduced the aortic plaque area, with the improvement being more pronounced in the BA high-dose group (**Figures 2B, C**). In terms of HE staining, we observed atherosclerotic changes in the aortas of mice in the HFB group, such as large areas of loose tissue, disorganised vascular endothelial cells and smooth muscle cells, and foam cell formation. Compared with the HFB group, we observed that a decrease in the area of loose tissue in the aortas of mice in the BAL group compared with the HFB group. In the BAH group, a more regular arrangement of endothelial and smooth muscle cells was observed in the mouse aorta compared to the HFB group, in addition to a reduction in the area of loose tissue. In conclusion, the above results indicate that BA can exhibit good anti- atherosclerosis effects (**Figures 2D, E**).

### 3.3 BA ameliorates gut microbiota disorders in AS co-depression mice

**Figure 3:**
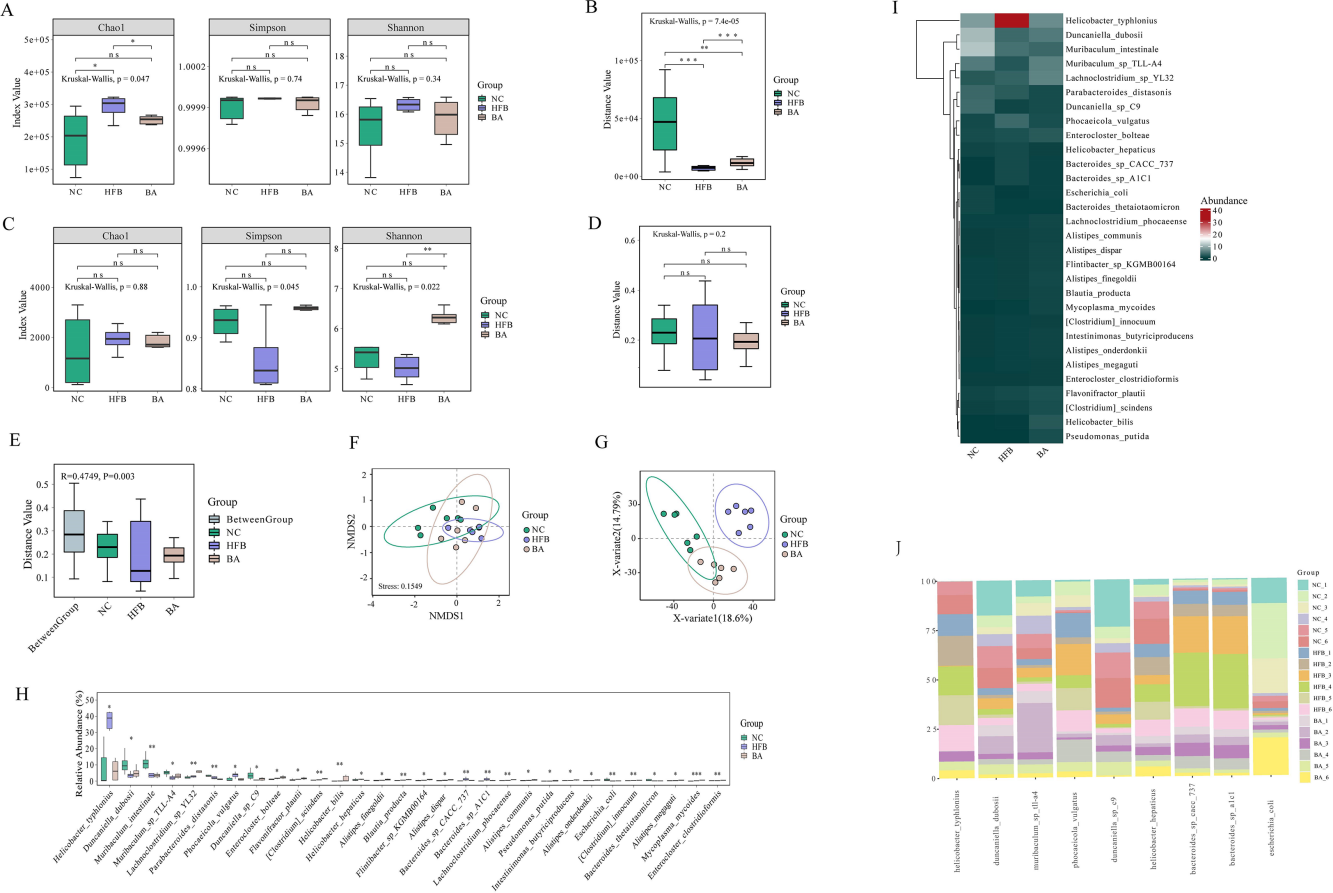
BA improves the gene, species, and functional diversity of the gut microbiota. **(A)** Gene Alpha diversity boxplot. **(B)** Gene Beta diversity boxplot. **(C)** Species Alpha diversity boxplot. **(D)** Species Beta Beta diversity boxplot. **(E)** Anosim analysis: Each sample’s distance is shown on the y-axis. The distances inside each group are shown by the remaining terms on the x axis, while the word “Between Group” shows the distances between groups. A higher intra-group similarity than an inter-group similarity is indicated by an R-value larger than 0; an R-value approaching 0 indicates no difference between inter-group and intra-group; R-value less than 0 indicates that the similarity between groups is greater than the intra-group. p is the value of the hypothesis test and P <0.05 indicates a significant difference between groups. **(F)** In the NMDS analysis of the scatterplots, it is usually considered that when stress < 0.2, the graphs have certain explanatory certain significance. **(G)** PLS-DA analysis scatterplot. **(H)** Species difference boxplot. Horizontal coordinates indicate species with statistically significant test differences, and vertical coordinates indicate the relative abundance of species. **(I)** Differential species abundance heatmap: every column denotes a sample, and every row denotes a species. **(J)** Differential species histogram, using different colours denoting various samples, the vertical axis showing the relative concentration of microorganisms and the horizontal axis listing species names at the species level. *, P < 0.05; **, P < 0.01; ***, P < 0.005; ****, P <0.001.

We used metagenomics sequencing to investigate the connection between gut microbiota and AS co-depression, first using gene Alpha diversity to describe the gene distribution of gut microbiota in NC, HFB, and BA group mice. Gene richness and evenness (**Figure 3A**), is commonly assessed by Chao1 index, Simpson’s index, and Shanno’s index. A larger Chao1 index represents more genes; a larger Shannon index indicates greater gene abundance and evenness in the sample; and a larger Simpson index value indicates greater genetic diversity. The results of the Chao1, Simpson, and Shanno indexes all showed that the index values in the HFB group increased compared to the NC group, whereas a decrease in the index values was observed after the intervention of BA. Specifically, the Chao1index demonstrated a significant drop (*P<0.05*) in the index value of the BA group relative to the HFB group and a significant rise (*P<0.05*) in the index value of the HFB group relative to the NC group. In order to depict the degree of sample dispersion within a group, the beta boxplot measures the distances between any two samples within the same group and performs a statistical test for the difference in distances between different groups. Beta diversity is defined as the degree of sample dispersion within the group (**Figure 3B**). The findings indicated that BA lessened the negative effects of HFB, the index value was considerably higher in the BA group compared to the HFB group *(P<0.001*) and the index value was notably lower in the HFB group compared to the NC group (*P<0.001*).

Subsequently, to explore the impact of BA on gut microbial species composition, we used species Alpha diversity as an indicator of species richness and evenness within habitats (**Figure 3C**). The Chao1 index results showed that the mean abundance of species in the BA intervention group tended to prefer the NC group over the HFB group, the mean number of species in the HFB group rose relative to the NC group. And the Simpson Index revealed that the HFB group had less community diversity than the NC group, and that the BA intervention had the opposite impact of the HFB’s negative consequences. The results of Shannon’s index revealed that the HFB group had lower species richness and evenness compared to the NC group, which was significant (*P<0.01*) improved after BA intervention. Species Beta diversity results showed (**Figure 3D**) that the HFB group had fewer discrete samples within the group than the NC group, while BA mitigated the adverse effects caused by high-fat feeding interventions. Anosim analysis (analysis of similarities) is a non-parametric test, which is used to test whether the between-group difference is greater than the within-group difference. An examination of Anosim determines the distance between two samples, arranges them in descending order of size, determines their rank, and finally computes the statistic. R tends to be near to 0 suggests that there is no difference between the within-group and between-group similarities, while R=0.4749 shows that the within-group similarity is larger than the between-group similarity (**Figure 3E**). Non-metric multidimensional scaling, or NMDS. A technique for data analysis called non-metric multidimensional scaling (NMDS) lowers research items (samples or variables) from a multi-dimensional space to a low-dimensional space so that they may be located, examined, and categorised while maintaining the original connections between the objects (**Figure 3F**). The findings demonstrated that the first, second, and third quadrants comprised the majority of the NC group samples distribution; the third and fourth quadrants comprised the majority of the HFB group samples distribution; and the samples from the BA intervention group were dispersed over the first, third, and fourth quadrants.

A discriminant analysis using partial least squares called PLS-DA (partial least squares discriminant analysis) was utilised to evaluate functional beta diversity, and the results showed that the samples of the NC group were mainly located in the second and third quadrants, the HFB group in the first quadrant, and the BA intervention group in the third and fourth quadrants and were closer to the NC group than to the HFB group (**Figure 3G**). The aforementioned partial metagenomics analyses’ results indicated that BA improved the genetic and species composition of the gut microbiota of AS co-depression mice and that the gut microbiota of these mice, when BA intervened, was more similar to the NC group than the model group. The advantages of BA in alleviating the gut microbiota of AS-co-depression mice were confirmed. Subsequently, to further screen for specific differential species significantly affected by BA, we performed a non-parametric statistical test (Wilcoxon/Kruskal-Willas) on the relative abundance of the species, screened out the 30 species-level microorganisms with *P<0.05* and the highest relative abundance, and plotted a box-and-line plot and a heatmap (**Figures 3H, I**). The relative abundance of nine of the species that showed a tendency to converge towards the NC group after BA intervention across samples was then presented in a bar chart (**Figure 3J**).

### 3.4 BA ameliorates brain lipid metabolism disorders in AS-co-depression mice

**Figure 4:**
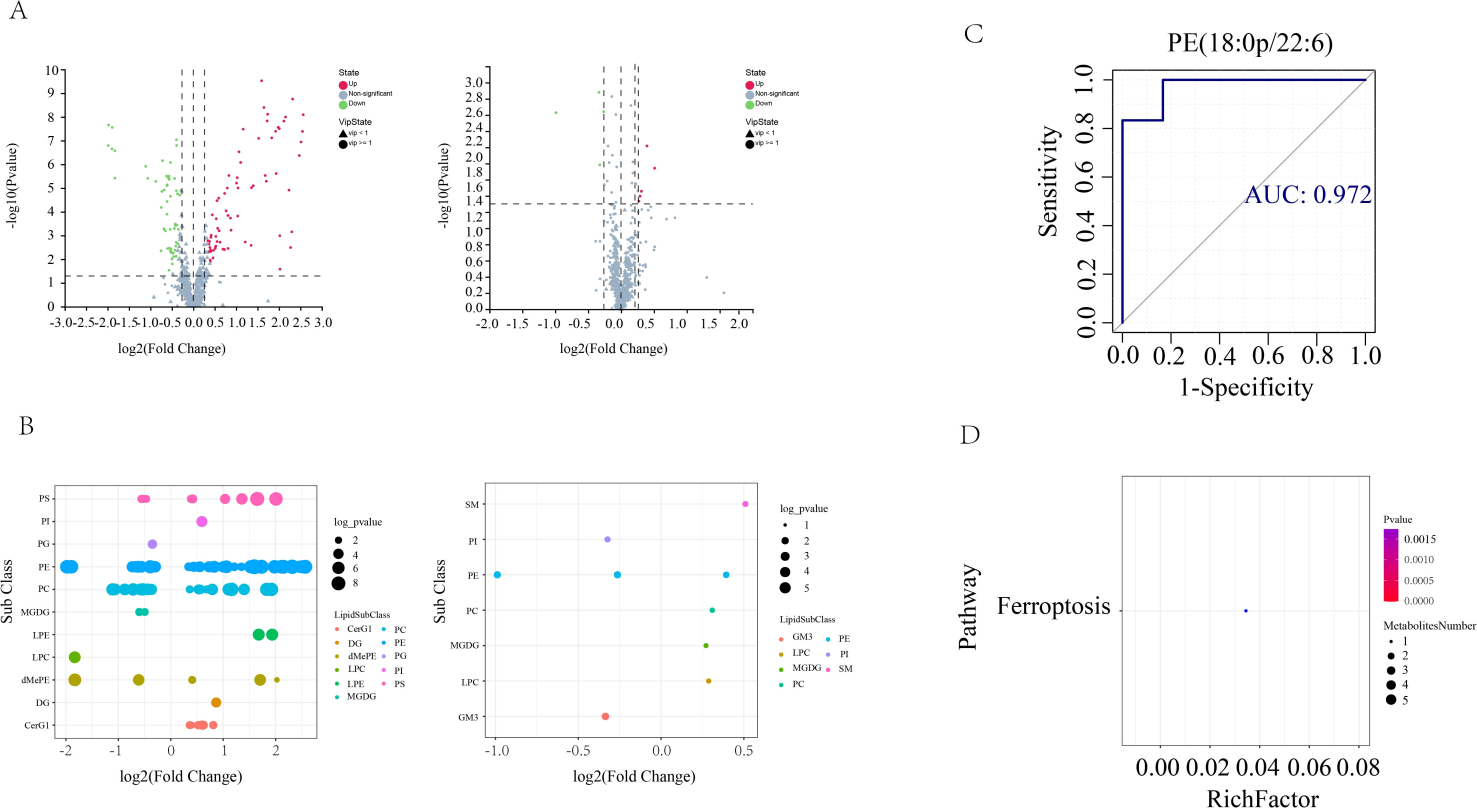
Hippocampal tissue differential lipid screening and analysis. **(A)** Volcano plots. Grey represents the nonsignificant metabolite, red the up-regulated significant difference metabolite, and blue the down-regulated significant difference metabolite. The metabolite with a VIP more than or equal to one is shown as a circle, the metabolite with a VIP less than one is shown as a triangle, and the insignificant metabolite is shown as grey (left: HFB group vs. NC group; right: BA group vs. HFB group). **(B)** Differential lipid bubble diagram of the HFB group vs. NC group (left) and the BA group vs. HFB group (right), the size of the dots is p following log10 conversion, each dot in the plots represents a differential lipid, and the colour of the dot relates to distinct lipid subclasses. **(C)** Differential lipids in hippocampus tissues are plotted using a ROC curve analysis; the area under the curve represents the AUC value. A higher AUC value suggests that the metabolite is a better candidate for use as a biomarker. **(D)** Enrichment plot of the KEGG metabolic pathway for differential lipids in hippocampal tissues. The number of distinct metabolites identified in this route is indicated by the size of the dots.

**Figure 5:**
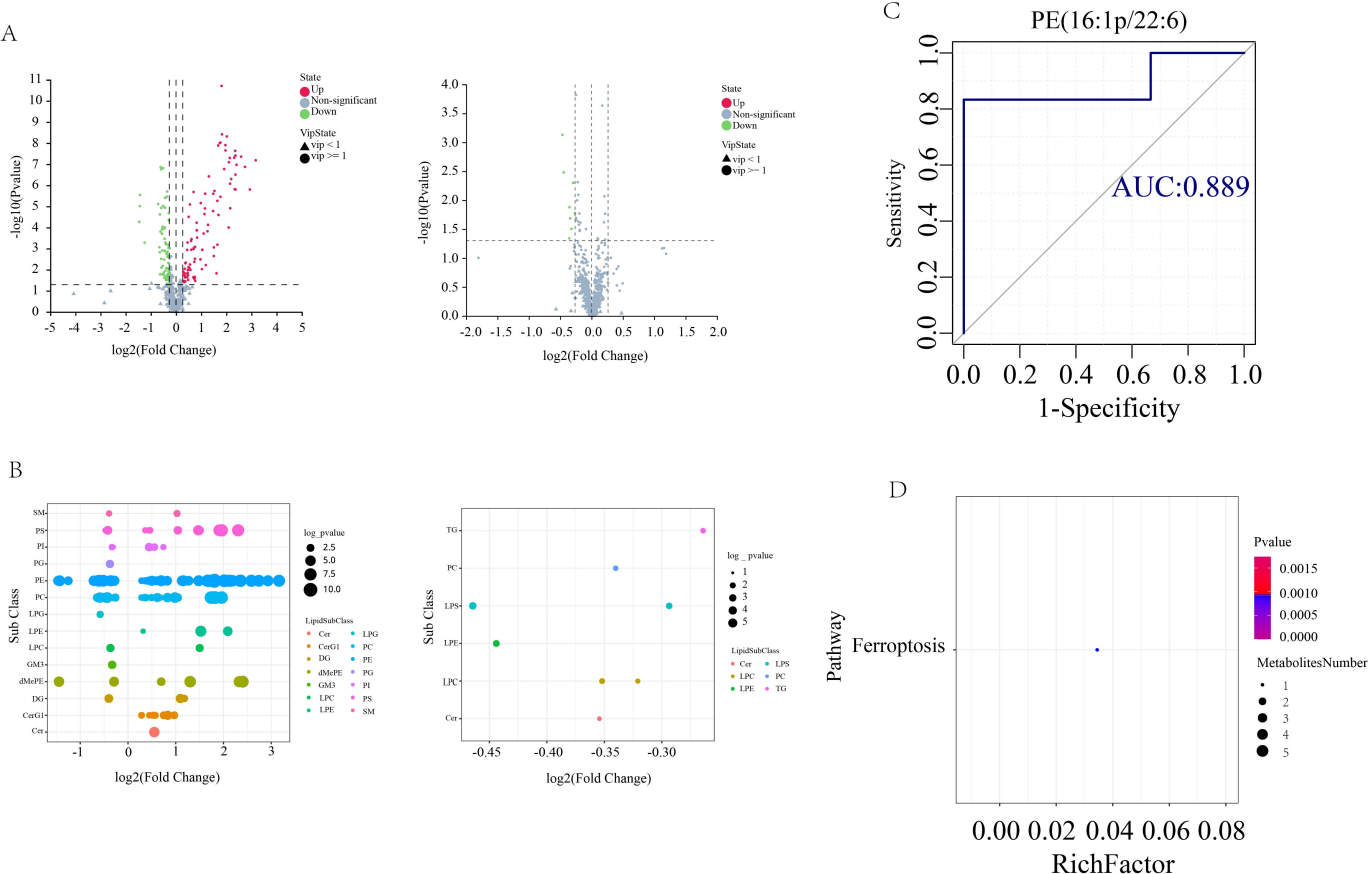
Prefrontal cortex tissue differential lipid screening and analysis. **(A)** Volcano plots. Grey represents the nonsignificant metabolite, red the up-regulated significant difference metabolite, and blue the down-regulated significant difference metabolite. The metabolite with a VIP more than or equal to one is shown as a circle, the metabolite with a VIP less than one is shown as a triangle, and the insignificant metabolite is shown as grey (left: HFB group vs. NC group; right: BA group vs. HFB group). **(B)** Differential lipid bubble diagram of the HFB group vs. NC group (left) and the BA group vs. HFB group (right), the size of the dots is p following log10 conversion, each dot in the plots represents a differential lipid, and the colour of the dot relates to distinct lipid subclasses. **(C)** Differential lipids in prefrontal cortex tissues are plotted using a ROC curve analysis; the area under the curve represents the AUC value. A higher AUC value suggests that the metabolite is a better candidate for use as a biomarker. **(D)** Enrichment plot of the KEGG metabolic pathway for differential lipids in prefrontal cortex tissues. The number of distinct metabolites identified in this route is indicated by the size of the dots.

In order to screen for lipids that may play an important role in AS co-depressive disorders, we performed metabolic lipidomics analyses of hippocampal and prefrontal cortex brain tissues, which are closely associated with depression. Hipocampal and prefrontal brain tissues were initially subjected to univariate analysis to look for distinct lipid compounds. As shown in **Figure 4**, 529 lipid molecules were discovered in the hippocampal tissue, of which 71 were significantly up-regulated and 56 were significantly down-regulated between the HFB and NC groups, and 5 were significantly up-regulated and 4 were significantly down-regulated between the BA and HFB groups (**Figure 4A**). 495 lipid molecules were discovered in the prefrontal cortex tissue, of which 94 were significantly up-regulated and 72 significantly down-regulated between the HFB and NC groups, and 8 significantly down-regulated differential lipids between the BA and HFB groups (**Figure 5A**). In order to dig deeper into the expression pattern of differential lipids, we analysed the content of different metabolic lipids. The findings demonstrated that the HFB group’s hippocampus tissue had different lipid subclasses than the NC group included PE, PC, PG, PI, PS, CerG1, DG, dMePE, LPC, LPE, and MGDG, and the differential lipid subclasses between the BA group and HFB group included PE, PI, SM, GM3, LPC, MGDG, and PC (**Figure 4B**). Differential lipid subclasses in prefrontal cortex tissue between the HFB group and the NC group included PE, PC, PG, PI, PS, SM, Cer, CerG1, DG, dMePE, GM3, LPC, LPE, and LPG; and between the BA group and the HFB group included PC, TG, Cer, LPC, LPE, and LPS (**Figure 5B**). In order to be able to screen for potential biomarkers to distinguish AS co-depression disease mice from healthy mice, we then performed a ROC curve analysis and found that the AUC value of PE (18:0P/22:6) in hippocampal tissues was 0.972, which was suitable as a potential biomarker for distinguishing between the BA group and the HFB group (**Figure 4C**). The AUC value of PE (16:1p/22:6) in the prefrontal cortex tissue was 0.889, suitable as a potential biomarker to differentiate the BA group from the HFB group (**Figure 5C**). Subsequently, we used the KEGG database to undertake metabolic pathway enrichment analysis of various lipids for the purpose of further recognise the metabolic pathways that saw notable modifications. The results showed that differential lipid PE (18:0/22:4) was significantly enriched in the ferroptosis pathway in both hippocampal and prefrontal cortex tissues (**Figures 4D** and **5D**).

### 3.5 Correlation analysis of different species with differential metabolites PEs

**Figure 6:**
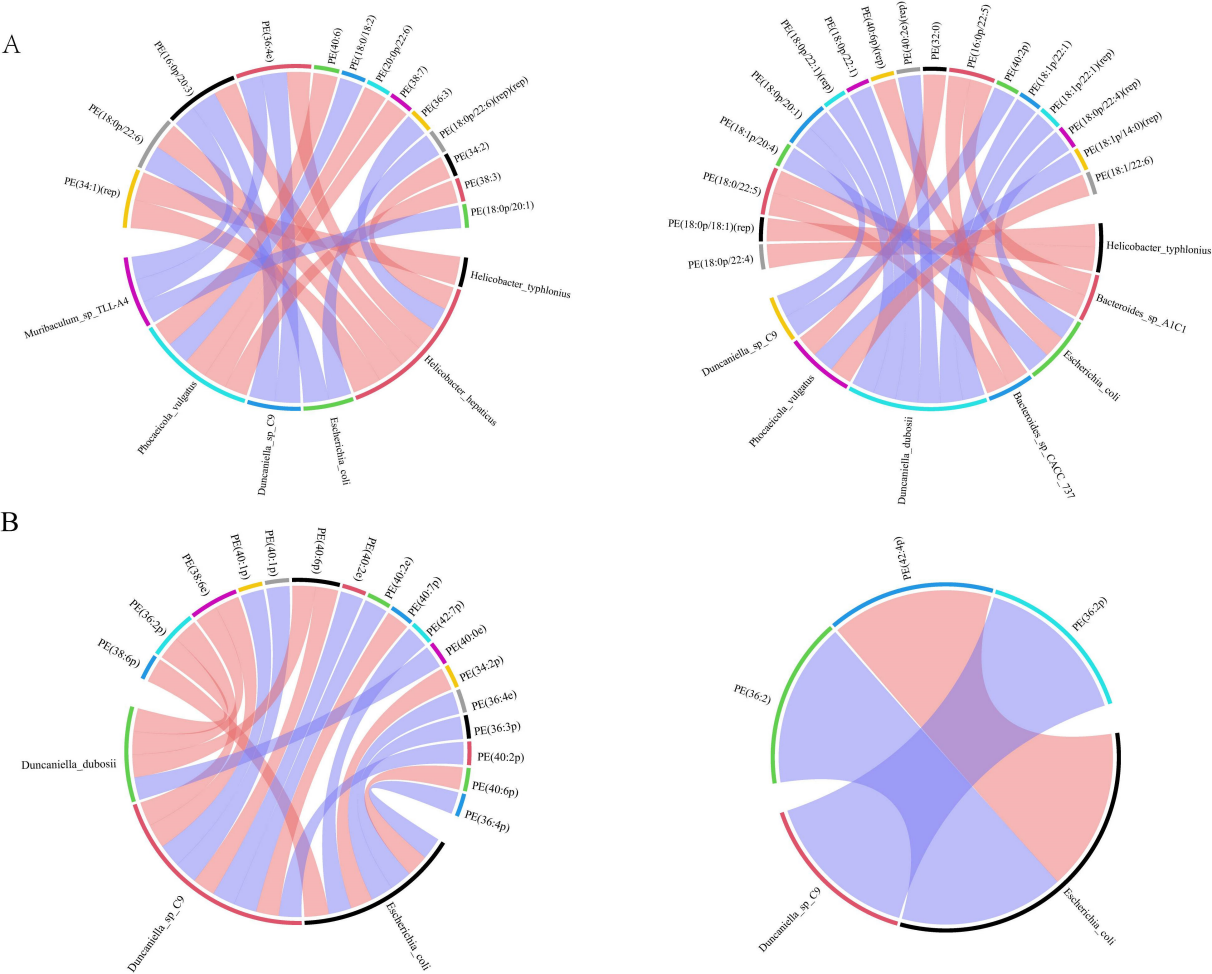
Correlation analysis between different species significantly affected by brain lipid PE and BA. We selected the top 20 correlated pairs with |r| > 0.7 and P < 0.05 for visualization. **(A, B)** On the outside of the circle are lipids and microbial taxa; within the circle are connecting lines that show the relationship between metabolites and microbial taxa, with blue and red hues denoting negative and positive association, respectively (A hippocampus, B prefrontal cortex, left: HFB group vs. NC group, right: BA group vs. HFB group).

To discover concerning the precise mechanism of the BA anti-AS co-depression and the connection between changed gut flora and lipid metabolism, we performed correlation analyses between the screened microorganisms significantly affected by BA and the differential metabolite PE. Spearman correlation analysis was used to assess the monotonic relationship between two continuous or sequential variables. The Spearman correlation coefficient between the different species significantly affected by BA and brain lipid PE was calculated, and the top 20 different species and brain lipid PE were selected to display as a chord diagram. As shown in **Figure 6**, among the nine species significantly affected by BA between the HFB and NC groups of hippocampal tissues, seven species were screened for strong correlation with PE, and six species were screened for strong correlation with PE between the BA and HFB groups. Four of these species, *Helicobacter typhlonius, Escherichia coli, Phocaeicola vulgatus*, and *Duncaniella* sp. *C9*, were strongly correlated with PE among the groups (**Figure 6A**). Between the HFB and NC groups of prefrontal cortex tissue, three of the nine species significantly affected by BA were screened for strong correlation with PE; between the BA and HFB groups, two species were screened for strong correlation with PE, and two of them, *Escherichia coli* and *Duncaniella* sp. *C9*, were found to be in strong correlation with PE between the groups (**Figure 6B**).

## 6 BA inhibited erastin-induced ferroptosis in HT-22 cells

**Figure 7:**
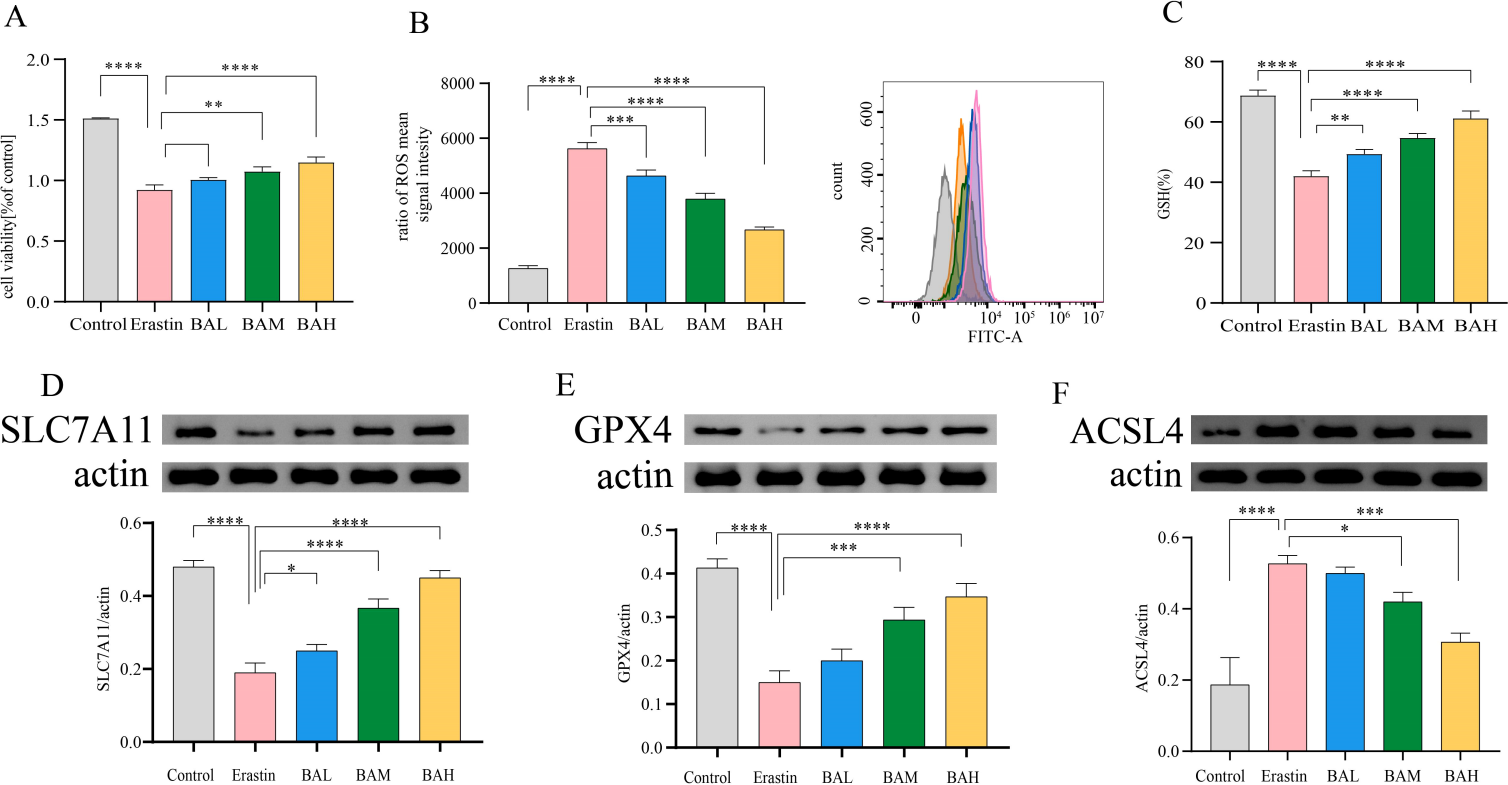
BA inhibited erastin-induced ferroptosis of HT-22 Cells. **(A)** Survival of HT22 cells under different action conditions was detected by the CCK-8 assay (N=3). **(B)** Intracellular ROS levels were measured using flow cytometry (N=3). **(C)** Intracellular GSH levels were measured by the trace reduced glutathione (GSH) assay kit (N=3). **(D–F)** Western blot was employed to determine SLC7A11, GPX4, and ACSL4 protein levels (N=3). All values are presented as mean ± SEM (N=6). *, P < 0.05; **, P < 0.01; ***, P < 0.005; ****, P < 0.001.

The effect of baicalin on Erastin-treated HT-22 cells was analysed to further confirm whether baicalin exerts neuroprotective effects by inhibiting ferroptosis. The CCK-8 test was used to determine the cell viability. The results, as shown in **Figure 7**, showed that the viability of HT-22 cells in the erastin group was 54.68% (*P < 0.0001*), and the cell viability in the different concentrations of BA intervention groups was 61.15%, 66.39% (*P < 0.01*), and 72.07% (*P < 0.0001*), respectively. The outcomes demonstrated that the cell survival rate was notably lower in the erastin group compared with the control group. Compared with the erastin group, the cell survival rate was notably higher in the 9μM and 27μM BA intervention groups, and there was a statistically significant difference, while there was no statistically significant difference in the 3 um BA intervention group. Therefore, BA pretreatment had a protective effect on erastin-injured HT-22 cells (the most pronounced protective effect was observed in the 27μM group) in a dose-dependent relationship (**Figure 7A**). ROS accumulation is one of the hallmarks of ferroptosis. In our study, we examined the expression level of ROS using flow cytometry, and the results demonstrated that erastin significantly promoted ROS production, whereas all three doses of BA significantly reduced ROS production, suggesting that BA could exert an anti-ferroptosis effect by ameliorating the cellular ROS accumulation (**Figure 7B**).

Erastin suppresses the cysteine (Cys)/glutamate (Glu) reverse transporter (system xc^-^), which causes lipid peroxides to build up and cause ferroptosis. This suppresses GSH production and lowers GPX4 activity (30). Thus, to further investigate the mechanism by which BA alleviates cellular ROS accumulation, we first quantified the concentration of GSH using a micro-reduced glutathione (GSH) assay kit. The results showed that erastin significantly *(P < 0.0001)* reduced intracellular GSH content, and this effect could be reversed by BA intervention in a dose-dependent relationship (**Figure 7C**). Subsequently, to confirm that BA was rescuing cell death through the amino acid metabolic pathway, we chose to perform a western blot assay on SLC7A11 (31), which exerts major transporter functional activity in system xc^-^, as well as GPX4 (32), a key regulator of ferroptosis. The findings demonstrated that BA enhanced HT-22 cell expression of SLC7A11 and GPX4 (**Figures 7D, E**). It also demonstrated the critical function BA plays in blocking the cysteine metabolic pathway’s induction of ferroptosis.

The PE lipid peroxidation reaction requires the participation of long-chain acyl-CoA synthetase family member 4 (ACSL4) and lysophosphatidylcholine acyltransferase 3 (LPCAT3) (24). ACSL4 is required to induce ferroptosis by accumulating oxidised cell membrane phospholipids. Therefore, to investigate whether BA also has the potential to inhibit ACSL4 from exerting anti-iron death effects. We performed Western blot for the content of ACSL4. The results showed that BA also had an inhibitory effect on the expression of ACSL4 in HT-22 cells (**Figure 7F**).

## 4 Discussion

AS co-depression is a serious hazard, the mechanism of co-morbidity is unclear, and clinical treatment faces great difficulties. Based on the fact that (1) lipid metabolism disorders caused by altered intestinal flora play a potentially important role in the pathogenesis of AS co-depression; (2) baicalin can regulate the intestinal flora and lipid metabolism to fight against AS and depression, and it is a potentially effective natural compound in preventing and ameliorating AS co-depression, thus we hypothesised that baicalin could play an anti-AS co-depressant role. The results of animal experiments showed that baicalin could effectively improve various indexes of AS co-depression mice, and the results of faecal metagenomic sequencing and lipidomics of hippocampus and prefrontal cortex showed that there were disorders of intestinal flora represented by *Helicobacter_typhlonius* and *Escherichia_coli* and disorders of lipid metabolism represented by PE in the AS co-depression model mice. The correlation analysis showed that the lipid metabolism disorders in the model mice were closely related to the intestinal flora disorders, and baicalin intervention could effectively improve the intestinal flora and lipid metabolism disorders in the AS co-depression mice. Metabolic pathway enrichment analysis showed that differential lipid PEs were all significantly enriched in the iron death pathway, and the results of our further *in vitro* cellular experiments indicated that baicalin inhibited erastin-induced iron death in the hippocampal neuronal cell line HT-22 in a variety of ways, such as by promoting the expression of SLC7A11, GSH, and GPX4, inhibiting the expression of ACSL4, and decreasing the cellular ROS, thereby protecting the neurons. The present study demonstrated that lipid metabolism disorders based on altered intestinal flora play an important role in the pathogenesis of AS co-depression, and baicalin exerts its Anti-atherosclerotic co-depression effects by improving intestinal flora and lipid metabolism disorders, and by inhibiting ferroptosis of neuronal cells, which are the several key links. It has important potential applications value.

The current clinical treatment plan for AS co-depression is still a simple superimposition of cardiovascular drugs and antidepressants, but studies have shown that routinely used cardiovascular medications such as angiotensin-converting enzyme inhibitors, β-blockers, aspirin, and diuretics do not reduce the risk of depression (33), and existing antidepressant drugs such as monoamine oxidase inhibitors, tricyclic antidepressants, and pentazocine re uptake inhibitors cause both cardiotoxicity such as malignant arrhythmias, lower blood pressure, and platelet dysfunction, as well as side effects such as insomnia, gastrointestinal dysfunction, and apathy in patients, forcing them to discontinue the medication if they are unable to tolerate it, and ultimately leading to clinical failure in more than 50% of patients (34, 35). The antidepressant effects of statins are also highly controversial, with some studies suggesting that statins may help reduce depressive symptoms by lowering blood lipids, while others have come to the exact opposite conclusion, suggesting that stations can have psychiatric adverse effects such as depression, anxiety, and suicide (36, 37). Therefore, the search for new effective programmes to prevent and improve AS co-depression is imminent.

Some natural compounds with both anti-AS and antidepressant activities contained in the diet are effective substances that deserve our close attention for potential prevention and improvement of AS co-depression, such as BA, which has been shown in previous studies to have good anti-AS activity as well as antidepressant effects, and it is inferred that BA may be a potentially effective natural compound (38, 39). Therefore, we first observed the effect of BA in AS co-depression mice. In the present study, we successfully constructed a mouse model of AS co-depression by combining a high-fat diet with bind intervention in ApoE^-/-^ mice. Mice with depressive-like behaviours often show multiple behavioural manifestations such as weight loss, reduced taste and smell functions, pleasure deficit-like behaviours, and decreased willingness to move or explore. Therefore, we chose to assess depressive-like behaviour in mice by body weight measurements, sucrose preference test, absent-field experiments and tail-hanging experiments: sucrose preference test is used to assess the animals’ preference for sweet substances; open field test detects the total distance the animals move in the open field environment within 5 minutes and the total time they move in the central area to assess the animals’ activity and willingness to explore; hanging tail test is used to assess the time the animals spend in the oppressive environment to produce the desperate immobility due to the inescapability. These are the currently accepted experiments to evaluate the depression-like behaviours in animals. The experimental results showed that compared with the NC group, the mice in the HFB had significantly lower body weight, decreased activity and willingness to explore, longer immobility state in the hanging tail experiment, and relatively lower preference for sugar and water, indicating that they had already shown significant depression-like behaviour. Compared with the HFB group, the depression-like behaviours of mice in the BA intervention group were significantly improved, indicating that BA can effectively improve the depression-like behaviours of mice in the AS co-depression model and has a good antidepressant effect (**Figure 1**).

Disorders of lipid metabolism are central and distinctive features that contribute to the development of atherosclerotic lesions. Therefore, we observed the effects of baicalin in regulating lipid levels in plasma and inhibiting aortic plaque formation in AS co-depression mice, and the results showed that BA significantly improved all abnormal lipid levels and reduced aortic plaque area in AS co-depression mice. In addition, in terms of HE staining, we not only observed that BA reduced the area of sparse tissue, but also observed that the arrangement of endothelial cells and smooth muscle cells was more regular compared to the HFB group. These results are encouraging and suggest that baicalin has a favourable anti-atherosclerotic effect and improves vascular morphology (**Figure 2**).

In recent years, with the deepening research on the regulatory role of intestinal flora, the interactions between intestinal flora and human brain and behaviour have been widely studied, and many potential molecular mechanisms have been gradually elucidated. A number of studies have demonstrated that alterations in the composition of intestinal flora are closely related to both the occurrence of AS and the development of psychiatric disorders (e.g., depression, anxiety) (40, 41), and some studies have shown that baicalin can improve AS and depression by regulating intestinal flora (22, 42). Metabolites of intestinal flora can affect the cardiovascular system and the central nervous system, including the immune system, the enteroencephalic nervous system and the endocrine system, through different pathways (43–45). Whether intestinal flora plays a key pivotal role in the pathogenesis of AS co-depression and whether baicalin exerts anti-AS co-depression effects based on gut flora alterations is a question of interest to us.

We used metagenomic sequencing to explore the relationship between gut flora and AS co-depression, and our results showed that BA improved the genetic and species diversity of AS mice, suggesting that BA was effective in improving the composition and distribution of gut microorganisms in AS co-depression mice at both the gene level and the species level. In addition, microbial gene function Beta diversity analysis also demonstrated higher similarity between the BA intervention group compared to the HFB group and the NC group. The beneficial effect of BA in improving the intestinal flora of AS co-depression mice was confirmed. Subsequently, we performed non-parametric statistical tests (Wilcoxon/Kruskal-Willas) on the relative abundance of species and screened the top 30 species-level microorganisms with p<0.05. A total of nine of these species showed a trend towards the NC group after BA intervention. In our study, we found that BA was able to significantly affect the two major Non-Helicobacter pylori Helicobacters in AS-co-depression mice, and they were significantly enriched in the gut microbiome of mice in the HFB group, which was significantly reduced by BA intervention. Previous studies have shown that bacterial DNA, such as Helicobacteraceae and Neisseriaceae, in the atherosclerotic plaques of symptomatic patients is more abundant compared with asymptomatic patients (46). The relative abundance of Lactobacillus, Helicobacter, and Desulfovibrio was considerably greater in hyperlipidaemic patients (47). These results echo our findings. Furthermore, the present study’s findings revealed that Helicobacter typhlonius was the species most severely influenced by BA in the experiment, and that it is a major illness trigger that encourages colitis by producing excessive amounts of TNF-α (48). In addition to causing colitis, Helicobacter hepaticus infection may raise the hepatic risk of contracting hepatitis C virus (HCV) infection (49, 50). Known as a chronic systemic and immune-mediated problem, atherosclerosis is linked to an increased risk of inflammatory bowel illness, myocardial infarction, venous thrombosis, and pulmonary thromboembolism, among other cardiovascular illnesses (51). Hepatitis C virus infection often becomes chronic, causing intrahepatic and extrahepatic inflammatory responses, leading to dysfunctions in cellular and humoral immunity, and encouraging the overexpression of factors that cause inflammation, increasing the risk of atherosclerotic lesions (52). Additionally, chronic hepatitis often causes metabolic disorders, including insulin resistance, which may also provide a risk for the development of AS (53).

Bacteroides sp. A1C1 and Bacteroides sp. CACC 737 were the two anamorphic bacilli significantly affected by BA in this study. The microbiological makeup of MDD sufferers’ guts from clinical studies is characterised by notable variations in the relative abundance of the phylum Firmicutes, Actinobacteria, and Bacteroidetes (54). Research on animals has disclosed that transplantation of Bacteroides fragilis, Bacteroides uniformis, and Bacteroides caccae into antibiotic-treated mice can negatively affect behaviour and neurogenesis via gut-brain metabolic signalling. Increased susceptibility to depression (55). This result is comparable to what we discovered that our study’s findings revealed a significant increase in the relative abundance of Bacteroides sp. A1C1 and Bacteroides sp. CACC 737 in the HFB group compared to the NC group, which was reversed by BA intervention. In addition, genera Bacteroides and Phocaeicola are species that have a significant function in the human colon, and of particular interest is Phocaeicola vulgatus, which, because of its prevalence in the human gut and capacity to break down a wide variety of heteropolysaccharides generated from plants, was originally categorised as a Bacteroides species. Despite the fact that Phocaeicola vulgatus has been identified as a hopeful probiotic for the treatment of illnesses linked to metabolic dysfunction (56). However, high levels of Phocaeicola vulgatus proteases, a protease that inhibits T-cell activity, have been discovered in ulcerative colitis patients’ intestines and are related to the illness’s level of sensitivity (57). Interestingly, we discovered that whereas the gavage of BA effectively decreased the relative abundance of Phocaeicola vulgatus in the stomach of ApoE^-/-^ mice, the relative abundance of Phocaeicola vulgatus in the gut microbiome of mice in the HFB group was dramatically raised.

Muribaculum sp TLL-A4 belongs to the genus Muribaculum, the family Muribaculaceae, and the phylum Bacteroidetes. Muribaculum is the dominant species that normally inhabits mice, with an average relative abundance of 1.9 percent, and mice and rodent intestines are the preferred habitat for species of the genus (58). Hongtao Xu et al. discovered that transplantation of faecal microbiota from dyslipidaemic donors induced a decrease in Muribaculum in humanised dyslipidaemic mice, resulting in a reduction in intestinal hypo-deoxycholic acid (HDCA) and a rise in the synthesis of bile acids, resulting in severe dyslipidemia (59). Ana-Sofa Medina-Larqué et al. found that oral administration of cranberry polyphenols (CP) and agavins (AG) was able to improve intestinal epithelial barrier function and repair epithelial barrier integrity by increasing the relative abundance of Muribaculum intestinal, promoting glycan degradation, and thus reducing the inflammation of metabolism and intestinal dysbiosis linked to obesity (60). Interestingly, BA successfully increased the relative abundance of Muribaculum sp TLL-A4 in AS-co-depression mice while ameliorating dyslipidemia resulting from a high-fat diet, which is in line with previous findings.

Duncaniella dubosii and Duncaniella sp. C9 were the two microorganisms of the genus Duncaniella significantly affected by BA in the current investigation. Prior research has demonstrated that elevated stress negatively impacts gut health and microbiome, as well as causing anxiety and depression. Research on animals revealed that emotional single lasting stress decreased the relative abundance of Duncaniella and caused adult male Swiss mice to exhibit gastrointestinal problems and a depression/anxiety-like phenotype (61). This is in line with our research, which found that a high-fat diet and binding stimulation led to a considerable drop in the relative abundance of Duncaniella dubosii and Duncaniella sp. C9 in the HFB group. This reduction was partially mitigated by the BA intervention.

Furthermore, we discovered in this study that the HFB group had a lower relative abundance of *Escherichia coli*. Whereas, the intervention by BA brought the content of Escherichia coli in mice closer to the NC group. *Escherichia coli* is a gramme-negative, parthenogenetic anaerobic bacterium that is a typical habitant of both humans’ and animals’ digestive tracts, accounting for approximately 1% of intestinal bacteria. Under normal conditions, the metabolic activity of *Escherichia coli* inhibits an increase in the digestive tract’s population of bacteria that break down proteins and reduces the negative consequences of products of protein breakdown on human health. *Escherichia coli* secretes colistin, which has a bactericidal effect. However, when the body’s bacterial microbiota is out of balance and immunity is compromised, Escherichia coli may migrate outside the intestinal tract, causing urinary tract infections, peritonitis, neonatal meningitis, sepsis, and other partially or systemically disseminated infections. It is therefore a conditionally pathogenic bacterium. *Escherichia coli* can synthesise vitamins B and K, and vitamin B, especially vitamin B12, plays a crucial part in the neurological system. A deficit in vitamin B12 can seriously harm nerve cells, causing them to lose both their natural shape and their ability to function, thus inducing a variety of neuropsychiatric disorders (62, 63). According to a recent study conducted on an elderly population, vitamin B12 deficiency was linked to a 70% higher risk of major depressive disorder in participants who were found to be deficient in the vitamin. 3,884 older persons were tested by the researchers for depression symptoms (64). Lower levels of B vitamins in the HFB group of mice in the present study may have resulted from lower levels of *Escherichia coli* K1, which caused the onset of depression, but further in-depth studies on the mechanism are still necessary. Additionally, there are some discrepancies between the current study’s findings and those of earlier research. Soo-Won Yun et al. found that gagging Escherichia coli K1 might cause depression and cognitive damage in germ-free animals and that this effect could be mediated by gavage of NK109 through the modulation of IL-1β expression, the gut microbiota, and gut-brain communication through the Vargas nerve to alleviate neuropsychiatric disorders (65). The discrepancy might be brought about by variations in sample selection and study methodology. Thus, more investigation is still required to completely comprehend the relationship between E. coli and depression.

AS is intimately associated with vitamin B. Hyperhomocysteinemia is the result of homocysteine building up in the body due to improper homocysteine metabolism caused by inadequate quantities of vitamins B12, B6, and folic acid (vitamin B9). Several domestic and international research have demonstrated that hcy is a significant cardiovascular disease threat (66). Significantly higher risk of peripheral vascular disease, cerebrovascular illness, and coronary heart disease is associated with hyperhomocysteinemia (67). Therefore, Hcy has become an important risk factor and predictor of cardio-cerebral and peripheral vascular diseases (68). Hcy is an intermediate metabolite of methionine, which is hazardous to vascular endothelial cells (69), causing vascular endothelial dysfunction or endangerment, lipid peroxidation, promoting platelet activation and generation, and increasing the adhesion of platelets in the blood, thus resulting in the creation of atherosclerotic plaques (70, 71). High levels of Hcy can stimulate excessive growth of arterial smooth muscle cells, interfere with the normal function of vascular smooth muscle, promote smooth muscle ageing, tissue fibrosis, and hardening, resulting in atherosclerosis and a dramatic increase in cardiovascular and cerebrovascular diseases (72). Consequently, it is clear that a high Hcy level poses a serious danger for the beginning of cardiovascular and cerebrovascular diseases.

In conclusion, the above findings suggest that BA can exert an anti-AS co-depressive impact by improving the gut microbiota (**Figure 3**).

Lipids are both a key factor in atherosclerotic lesions (16) and an important component of neuronal cell membranes within the brain, which is essential for the survival and transmission of neurones (17, 18), and unbalance in lipid metabolism has been demonstrated a metabolic feature of depression patients (73), whereas studies on the lipid metabolism in the brains of AS co-depression mice are few. In addition, it is not clear whether BA can ameliorate the AS co-depression mice’s brain lipid metabolism disorders. Thus, in order to screen for lipids that may play an important role in AS co-depressive disorders, we performed metabolic lipidomics analyses of hippocampal and prefrontal cortex brain tissues, which are closely associated with depression. We found that differential lipids, represented by PE, were significantly differentially altered in both hippocampal and prefrontal cortex tissues of AS co-depression mice in the present study. Among them, PE (18:0P/22:6) in hippocampal tissue as well as PE (16:1p/22:6) in prefrontal cortex tissue were suitable as potential biomarkers to distinguish the BA group from the HFB group, respectively. It is also noteworthy that differential lipid PE (18:0/22:4) in both hippocampal and prefrontal cortex tissues were significantly enriched in the ferroptosis pathway in the present study. Suggesting that ferroptosis pathway in our neuronal cells may assume an important role in the pathogenesis of AS co-depression mice (**Figure 4**, **Figure 5**).

Research has indicated that the connection between host metabolism and the gut microbiota is crucial in the reason for depression (74) and that by means of themselves or their byproducts, gut microorganisms can take part in the host’s metabolism, which are closely related to the metabolic processes of lipids (14, 15). These are key factors in atherosclerotic lesions (16) and in the start and progression of depression (75). Therefore, it is suggested that baicalin plays an anti-AS co-depression role by improving intestinal flora and lipid metabolism disorders. To discover concerning the precise mechanism of BA anti-AS co-depression and the connection between changed gut flora and lipid metabolism, we performed correlation analyses between the screened microorganisms significantly affected by BA and the differential metabolite PE. The results indicate that species significantly affected by BA intervention are closely related to brain lipid PE metabolism, further suggesting that BA may affect brain lipid PE metabolism through the intestinal flora(**Figure 6**). The current study concluded that PE oxidation is closely related to ferroptosis, in which PE (18:0/20:4 and 18:0/22:4) containing trioxo arachidonic acid (AA) - and adrenoic acid (AdA) are the foremost and sensitive substrates for lipid peroxidation in the ferroptosis signalling(Kagan et al., 2017), and that PE can be converted to long-chain acyls in the presence of lysophosphatidyltransferase 3 (LPCAT3). Coenzyme A (CoA) synthase 4 (ACSL4) -catalysed reacylation of synthesised lipoyl CoA, resulting in PE-AA and PE-AdA that can be oxidised by lipoxygenases (LOXs) to generate lipid peroxides that trigger ferroptosis (76).

Scholarly interest in depression caused by ferroptosis in central nervous systems has grown in the last several years. In addition to directly inducing neuronal cell death, ferroptosis can also lead to depression through 1) activation of the CNS immune-inflammatory response; 2) inducing mitochondrial dysfunction; 3) leading to a brain stress response through gut microbial imbalance; and 4) affecting cellular autophagy (77). Recently, Jingjing Chen et al. discovered that ferroptosis is a major factor in the pathophysiology of depression and that genes linked to ferroptosis are not only good biomarkers for identifying depression individuals from healthy controls but are also intimately associated with the immune system’s reaction to depression (78), whereas antidepressant effects can be exerted through inhibition of the ferroptosis signalling pathway (79, 80). Therefore, we hypothesised that the alteration of gut microbiota in mice affecting PE metabolism in brain tissue and thus triggering neuronal cell ferroptosis may be a molecular mechanism throughout the pathology of AS co-depression and that BA can exert anti-AS co-depressant effects at multiple points both by improving gut microbiota and lipid metabolism disorders, as well as by directly crossing the blood-brain barrier and inhibiting neuronal cell ferroptosis (81).

The central mechanism of ferroptosis is lipid peroxide accumulation (82). In the present study we found significant differential alterations in brain tissue PE in AS co-depression mice based on lipidomic analysis and annotated to the ferroptosis pathway. To further confirm the anti-ferroptosis effect of BA, we selected the hippocampal neuronal cell line HT-22 to establish an ferroptosis cell model using erastin treatment for 24 h. In addition, literature research has found that baicalin can penetrate the blood-brain barrier directly act on nerve cells in addition to regulating intestinal microbiota and lowering blood lipids. Therefore, in order to verify that BA can directly act on nerve cells to exert a protective effect, we selected the hippocampal neuronal cell line HT-22 to establish a ferroptosis cell model with Erastin treatment for 24 h, and pretreated HT-22 cells with different concentrations of BA (3 μM, 9 μM, 27 μM) for 2 h, and then co-incubated with Erastin (10 μM) for 24 h, and detected cell viability with CCK-8 test. The detection of ROS expression levels by flow cytometry. The results showed that BA rescued the damage caused by Erastin to HT-22 cells, effectively reduced the production of ROS, and exerted a significant anti-ferroptosis effect.

Glutathione (GSH) is central to abnormal amino acid metabolism leading to ferroptosis. GPX4 is an antioxidant enzyme that interacts with GSH to reduce toxic lipid peroxides to non-toxic fatty alcohols, and plays an important role in cellular antioxidant defence and inhibition of cellular ferroptosis. Erastin suppresses the cysteine (Cys)/glutamate (Glu) reverse transporter (system xc^-^), which causes lipid peroxides to build up and cause ferroptosis. This suppresses GSH production and lowers GPX4 activity (30). Therefore, in order to further explore the mechanism by which BA alleviates ROS accumulation in cells, we performed quantitative detection of GSH and western blot detection of SLC7A11 (31), which plays a major transport function in system xc^-^, and GPX4 (32), a key regulator of ferroptosis. The results showed that BA increased the expression of SLC7A11 and GPX4 in HT-22 cells. It was further confirmed that BA played an important role in the intervention of ferroptosis induced by cysteine metabolic pathway.

The PE lipid peroxidation reaction requires the participation of ACSL4 and LPCAT3 (24). ACSL4 is required to induce ferroptosis by accumulating oxidised cell membrane phospholipids. Catalysed by ACSL4 and LPCAT3, PE is ultimately oxidised to lipid hydroperoxides, which act as ferroptosis signals. Thus, inhibition of ACSL4 and LPCAT3, which makes cells less sensitive to ferroptosis, is a crucial pharmaceutical target for the management of illnesses associated with ferroptosis. To investigate whether BA also has the potential to target ACSL4 for anti-ferroptosis effects. We performed a Western blot assay on the content of ACSL4. The results showed that BA also had an inhibitory effect on ACSL4 expression in HT-22 cells (**Figure 7**).

## 5 Conclusion

**Figure 8:**
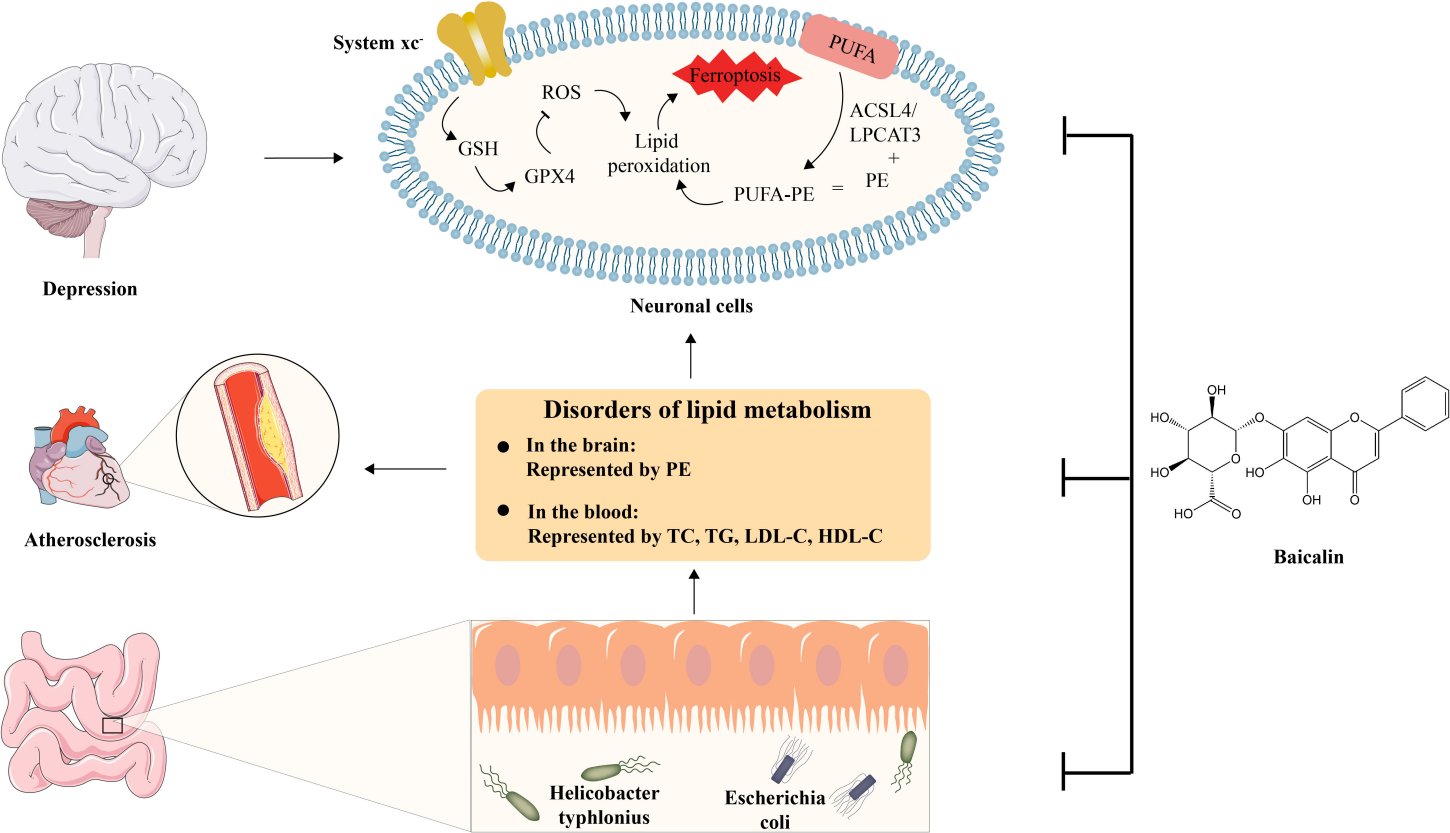
Schematic diagram of the anti-AS co-depression effect of baicalin. BA exerts its anti-AS co-depressant effects by improving several key links, including 1) intestinal dysbiosis, 2) disturbance of lipid metabolism represented by TC, TG, LDL-C, HDL-C in the blood and PE in the brain, and 3) Inhibition of Ferroptosis in neuronal cell.

This study investigated the mechanisms of action and anti-AS co-depressive effects of BA. The outcomes of behavioural tests including the tail suspension, open field, and sucrose preference tests demonstrated that baicalin might ameliorate depressive-like behaviour in AS co-depression mice. Lipid biochemical detection and histological tests, including oil red O and HE staining, demonstrated that baicalin may treat atherosclerotic plaque lesions and lower the hyperlipidemia level in AS co-depression animals. The results of mice faecal metagenomics sequencing showed that there were gut microbiota disorders represented by *Helicobacter typhlonius* and *Escherichia coli* in AS co-depression mice, and the results of mice hippocampus and prefrontal cortex lipidomics showed that there were lipid metabolism disorders represented by PE in AS co-depression mice. The results of the correlation analysis demonstrated that the disruption of intestinal flora and lipid metabolism in AS co-depression animals were closely associated, and that baicalin intervention could effectively alleviate both of these disturbances in these mice. Differential lipid PE was substantially enriched in the ferroptosis pathway in both the prefrontal cortex and the hippocampus tissues, according to metabolic pathway enrichment analysis of various lipids relied on the KEGG database. Further *in vitro* cellular experiments showed that in the hippocampus neuronal cell line HT-22, BA may prevent erastin-induced ferroptosis by upregulating the expression of SLC7A11, GSH, and GPX4, suppressing the protein of ACSL4, and reducing cellular ROS in a number of other ways (**Figure 8**).

The present research showed that lipid metabolic abnormalities based on modifications in gut microbiota possess a major impact on the pathophysiology of AS co-depression, and BA, with its biological activities of improving gut microbiota and lipid metabolism disorders and inhibiting neuronal ferroptosis, can effectively prevent and improve AS co-depression, which is of important potential application value, providing more experimental data support for medicinal plants to guide human health.

## Data Availability

The original contributions presented in the study are publicly available. Metagenomics data has been uploaded to the NCBl database (SRA data: PRJNA1247133. Lipidomics data has been uploaded to China National Center for Bioinformation (Item number: PRJCA038301 (The name of resource: OMIX009713, OMIX009727, OMIX009729).
