# Supplementary material for: Comprehensive metagenomic and lipidomic analysis showed that baicalin could improve depressive behaviour in atherosclerotic mice by inhibiting nerve cell ferroptosis
Source: Front Immunol. 2025 Sep 5;16:1599570. doi: 10.3389/fimmu.2025.1599570 (PMC12446369; doi:10.3389/fimmu.2025.1599570)
Supplement: Supplementary file 1 [file DataSheet1.zip › ╘¡╩╝╩2╛▌╔╧┤1⁄2/raw data.docx]

The following describes the data upload result：

Metagenomics data has beenuploaded to the NCBl database（SRA data: PRJNA1247133，Temporary Submission ID: SUB15236206）

<https://www.ncbi.nlm.nih.gov/>

Lipidomics data has been uploaded to China National Center for Bioinformation(Item number: PRJCA038301，The name of resource：OMIX009713，OMIX009727，OMIX009729）

<https://www.cncb.ac.cn/>
