# Supplementary material for: Comprehensive metagenomic and lipidomic analysis showed that baicalin could improve depressive behaviour in atherosclerotic mice by inhibiting nerve cell ferroptosis
Source: Front Immunol. 2025 Sep 5;16:1599570. doi: 10.3389/fimmu.2025.1599570 (PMC12446369; doi:10.3389/fimmu.2025.1599570)

	Sample Name	Subset Name	Count
	-3.fcs	Ungated	11224
	BAL -3.fcs	Ungated	10871
	BAM -1.fcs	Ungated	10179
	-2.fcs	Ungated	10130
	BAH -3.fcs	Ungated	10763

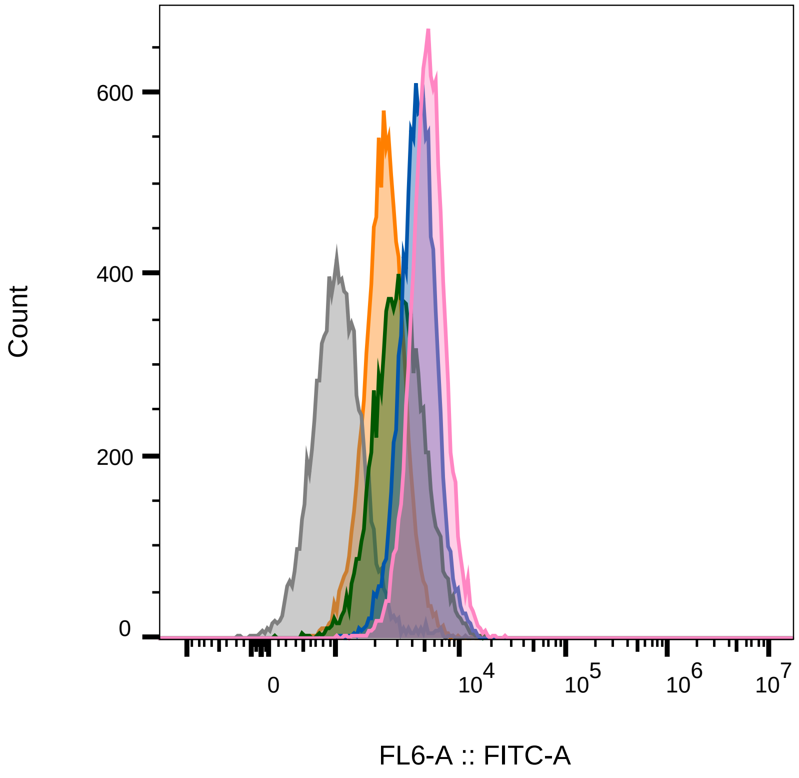

Supplement: Supplementary file 1 [file DataSheet1.zip › ╘¡╩╝╩2╛▌╔╧┤1⁄2/6 Cell experiments/B ROS/ROS(1).pdf]
