# Supplementary material for: Comprehensive metagenomic and lipidomic analysis showed that baicalin could improve depressive behaviour in atherosclerotic mice by inhibiting nerve cell ferroptosis
Source: Front Immunol. 2025 Sep 5;16:1599570. doi: 10.3389/fimmu.2025.1599570 (PMC12446369; doi:10.3389/fimmu.2025.1599570)
Supplement: Supplementary file 1 [file DataSheet1.zip › ╘¡╩╝╩2╛▌╔╧┤1⁄2/6 Cell experiments/DEF WB/Original Images.docx]

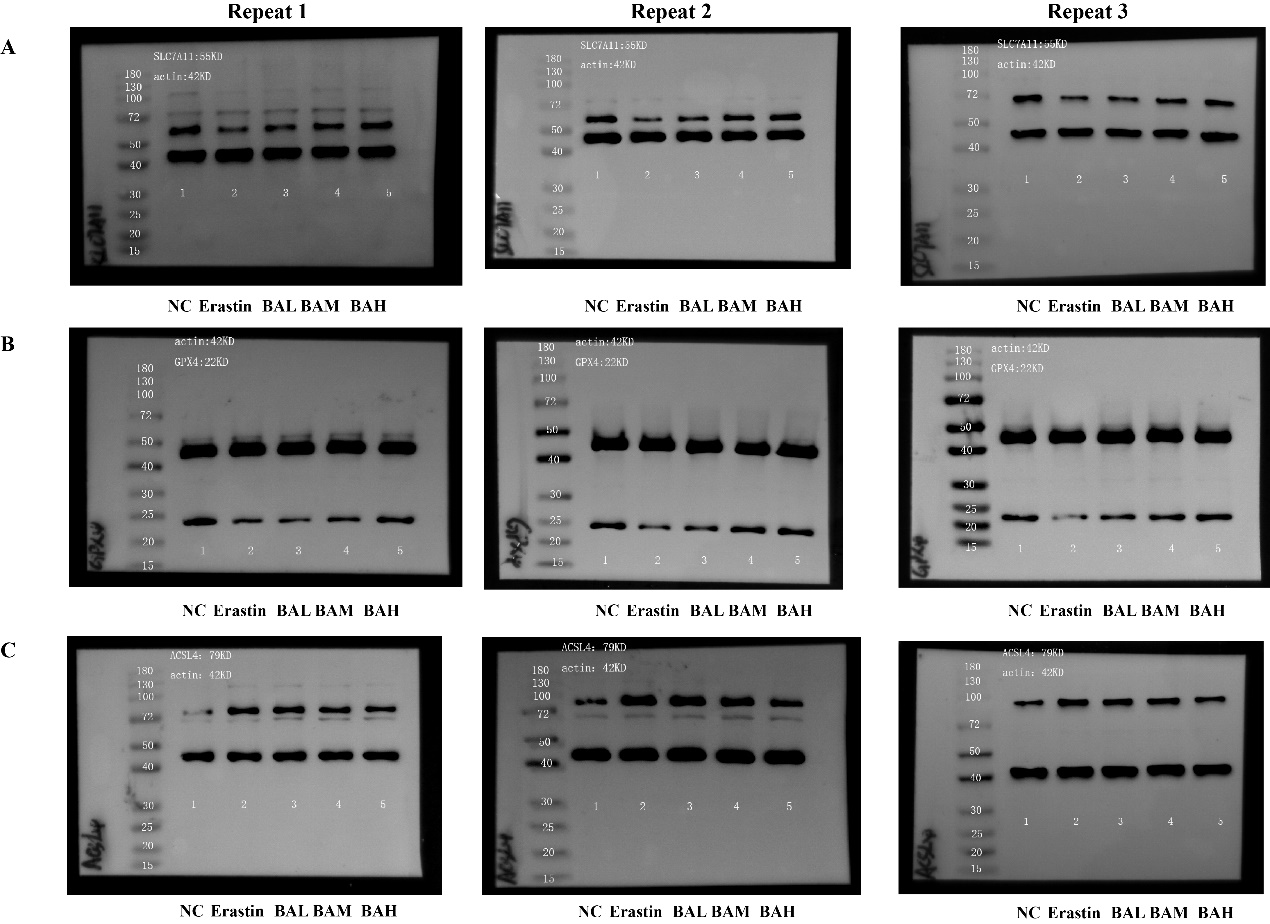


Fig. 1 The original Western Blots images of SLC7A11、GPX4 and ACSL4

(A) represents the data shown in Fig. 7D; (B) represents the data shown in Fig. 7E; (C) represents the data shown in Fig. 7F.
