# Supplementary figures and images for: Comprehensive metagenomic and lipidomic analysis showed that baicalin could improve depressive behaviour in atherosclerotic mice by inhibiting nerve cell ferroptosis
Source: Front Immunol. 2025 Sep 5;16:1599570. doi: 10.3389/fimmu.2025.1599570 (PMC12446369; doi:10.3389/fimmu.2025.1599570)

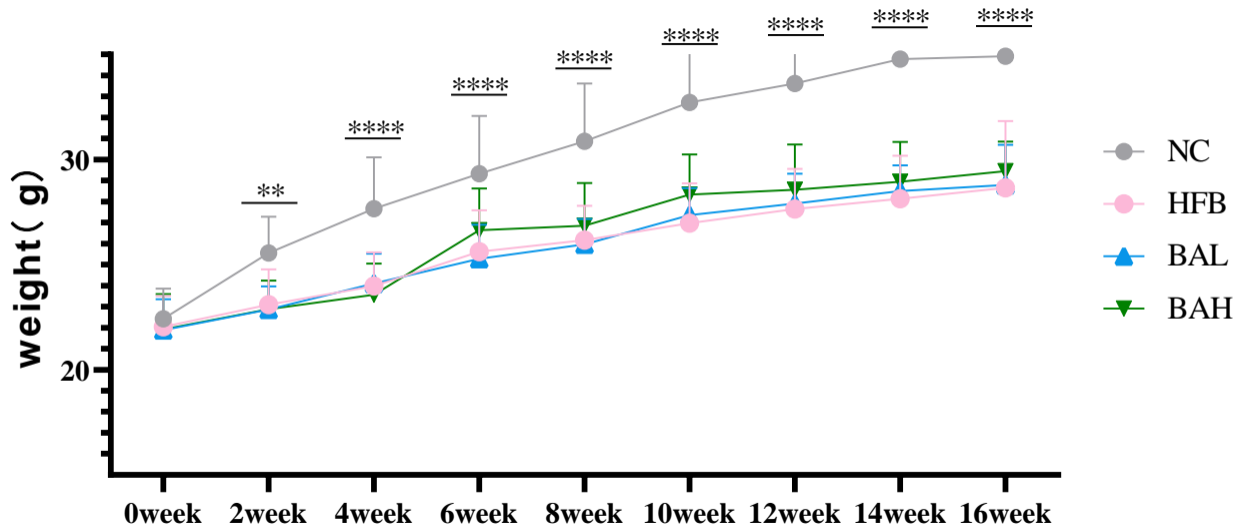

Supplement: Supplementary file 1 [file DataSheet1.zip › ╘¡╩╝╩2╛▌╔╧┤1⁄2/1 behavior data/Body Weight.pdf]

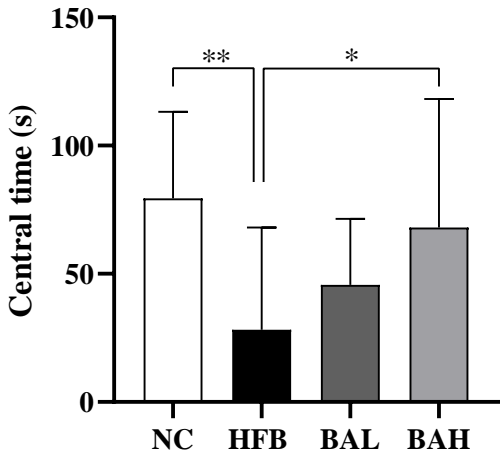

Supplement: Supplementary file 1 [file DataSheet1.zip › ╘¡╩╝╩2╛▌╔╧┤1⁄2/1 behavior data/Open field test 2.pdf]

# Open field test

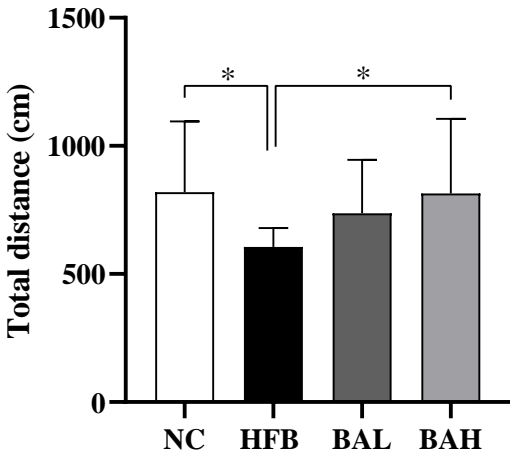

Supplement: Supplementary file 1 [file DataSheet1.zip › ╘¡╩╝╩2╛▌╔╧┤1⁄2/1 behavior data/Open field test.pdf]

## Sucrose preference test

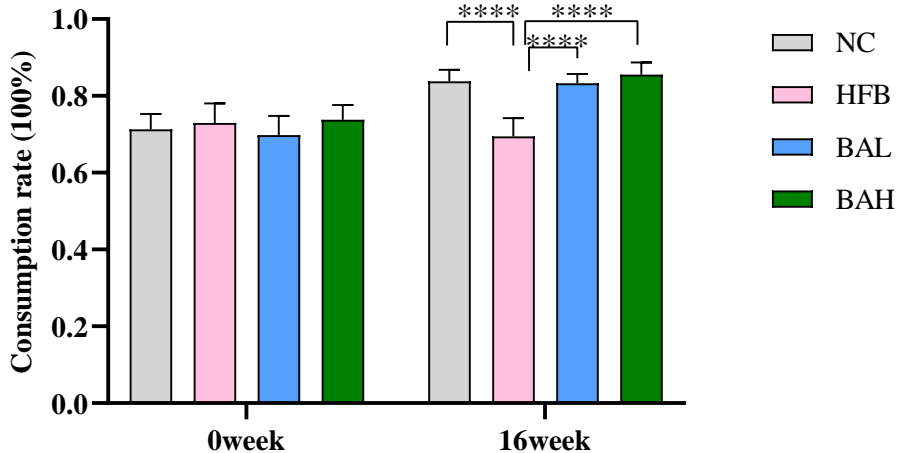

Supplement: Supplementary file 1 [file DataSheet1.zip › ╘¡╩╝╩2╛▌╔╧┤1⁄2/1 behavior data/Sucrose preference test.pdf]

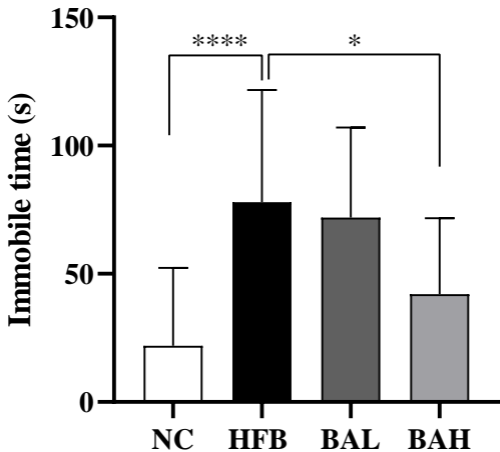

Supplement: Supplementary file 1 [file DataSheet1.zip › ╘¡╩╝╩2╛▌╔╧┤1⁄2/1 behavior data/Tail suspension experiment.pdf]

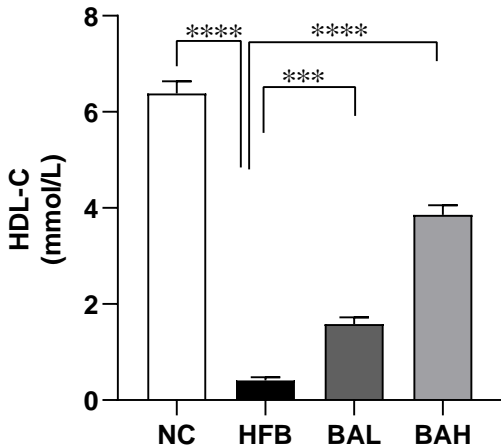

Supplement: Supplementary file 1 [file DataSheet1.zip › ╘¡╩╝╩2╛▌╔╧┤1⁄2/2 Indicators of atherosclerosis/A Blood lipid levels/HDL-C.pdf]

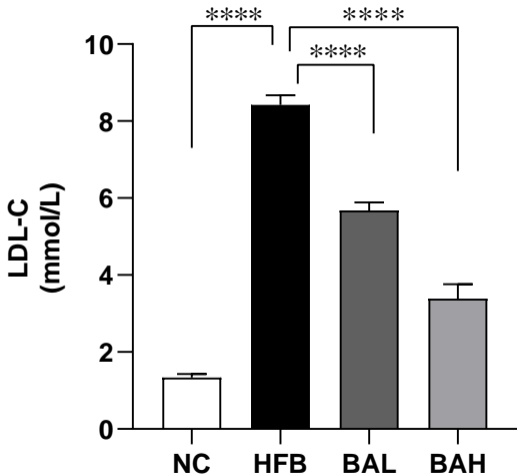

Supplement: Supplementary file 1 [file DataSheet1.zip › ╘¡╩╝╩2╛▌╔╧┤1⁄2/2 Indicators of atherosclerosis/A Blood lipid levels/LDL-C.pdf]

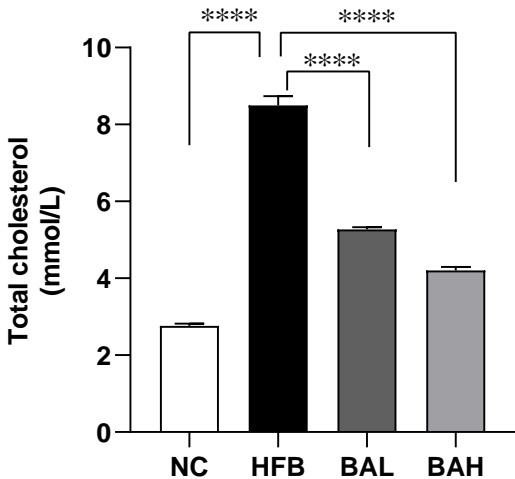

Supplement: Supplementary file 1 [file DataSheet1.zip › ╘¡╩╝╩2╛▌╔╧┤1⁄2/2 Indicators of atherosclerosis/A Blood lipid levels/TC.pdf]

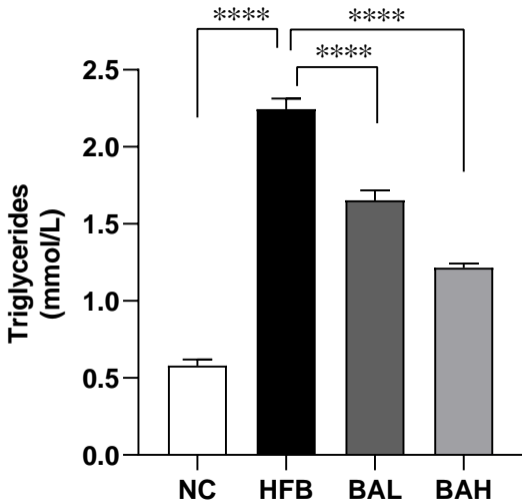

Supplement: Supplementary file 1 [file DataSheet1.zip › ╘¡╩╝╩2╛▌╔╧┤1⁄2/2 Indicators of atherosclerosis/A Blood lipid levels/TG.pdf]

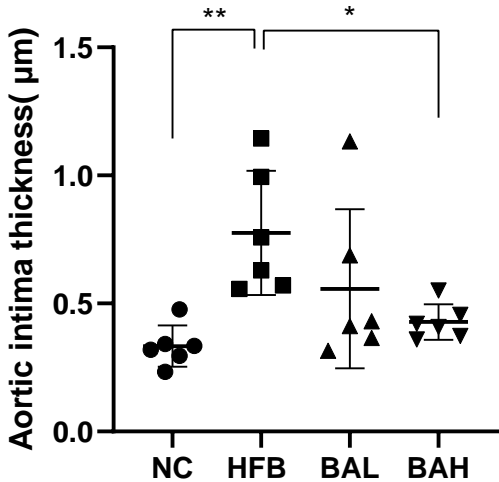

Supplement: Supplementary file 1 [file DataSheet1.zip › ╘¡╩╝╩2╛▌╔╧┤1⁄2/2 Indicators of atherosclerosis/BC HE/HE.pdf]

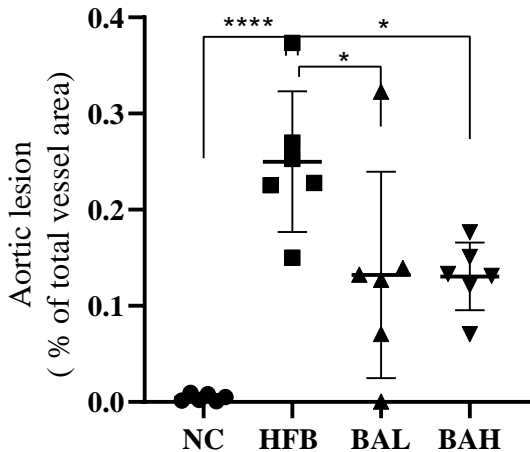

Supplement: Supplementary file 1 [file DataSheet1.zip › ╘¡╩╝╩2╛▌╔╧┤1⁄2/2 Indicators of atherosclerosis/DE Oil Red O/Oil Red O.pdf]

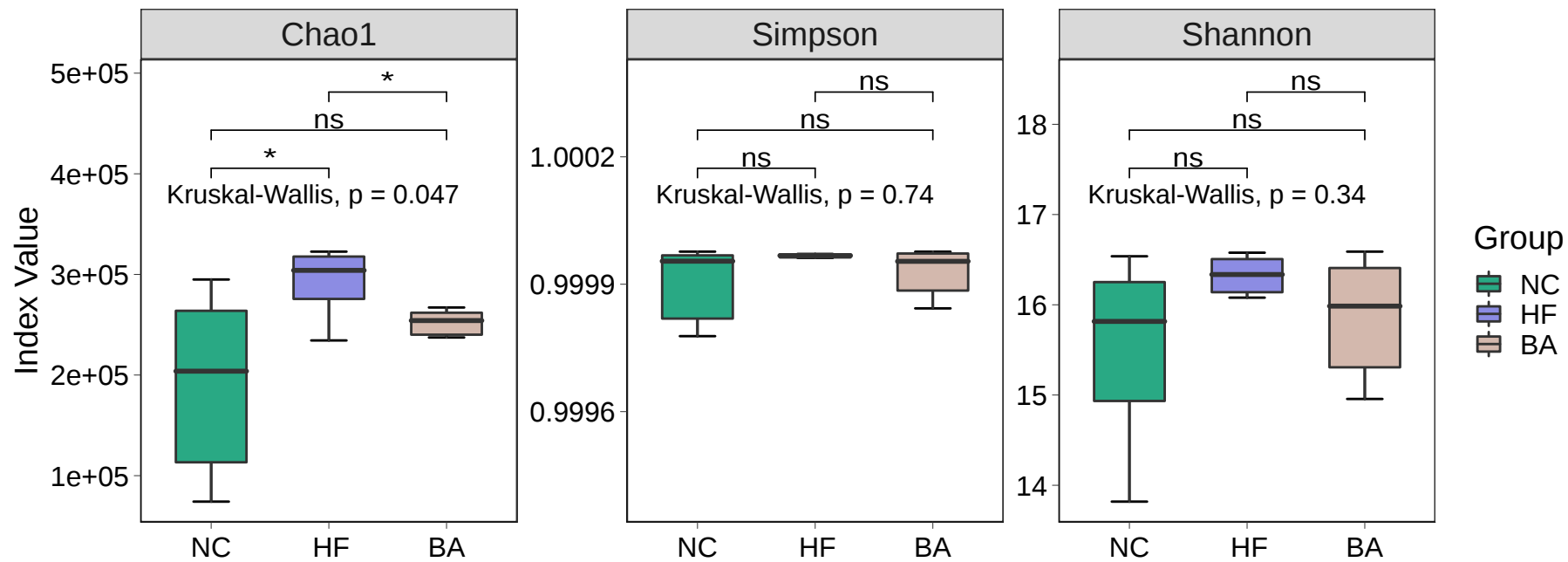

Supplement: Supplementary file 1 [file DataSheet1.zip › ╘¡╩╝╩2╛▌╔╧┤1⁄2/3 Metagenomics/A Gene Alpha diversity/Gene Alpha diversity_202504021935.pdf]

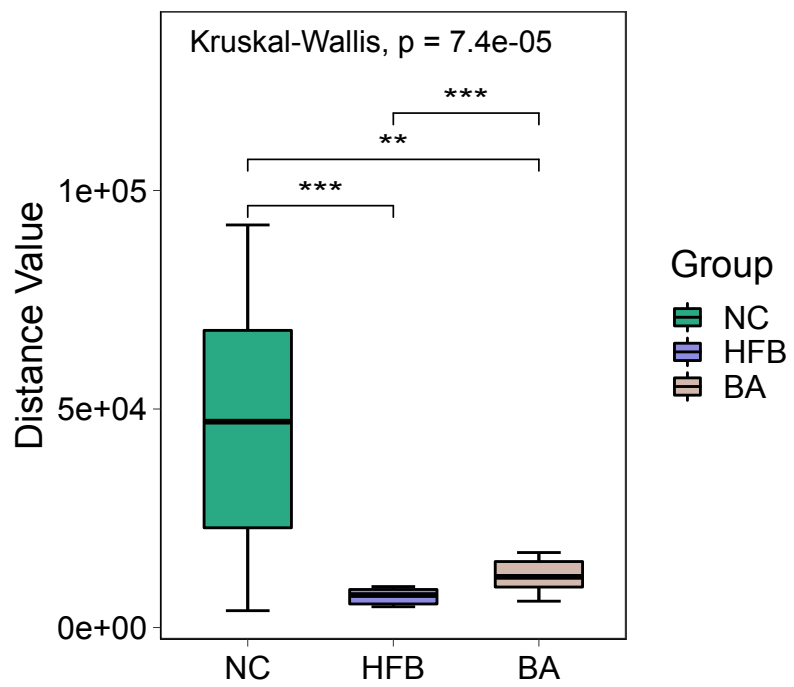

Supplement: Supplementary file 1 [file DataSheet1.zip › ╘¡╩╝╩2╛▌╔╧┤1⁄2/3 Metagenomics/B Gene Beta diversity/Gene Beta diversity.pdf]

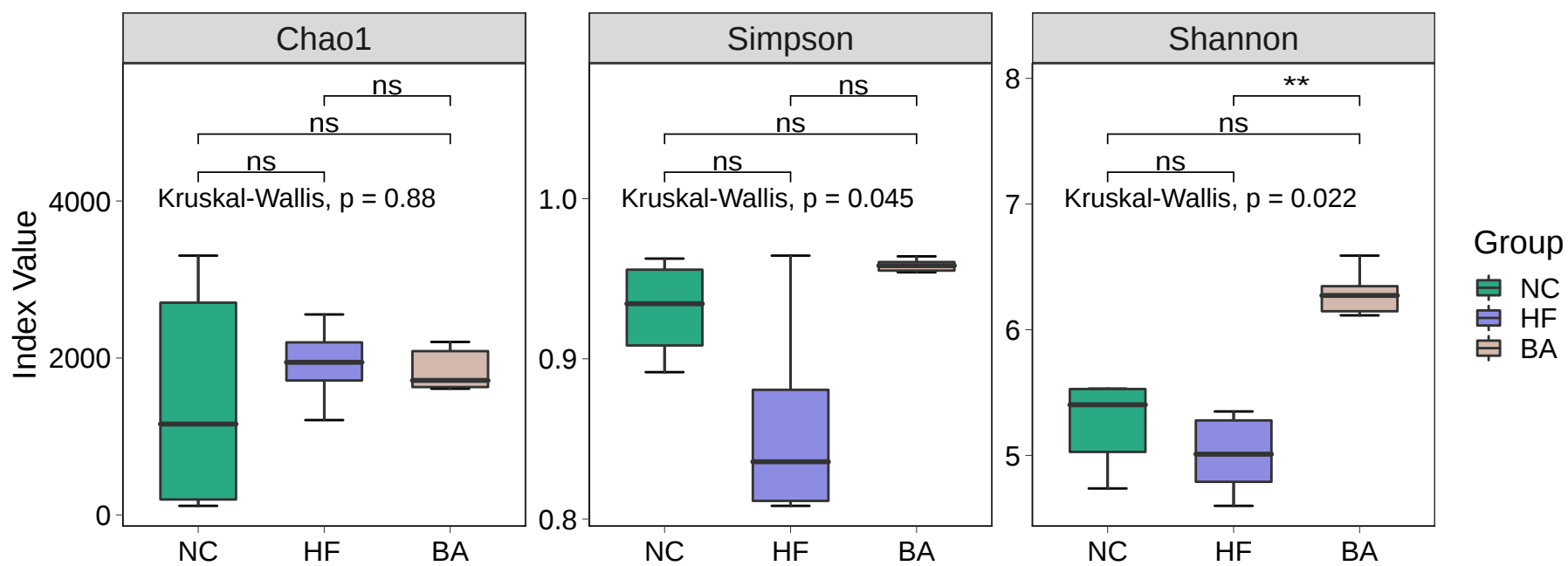

Supplement: Supplementary file 1 [file DataSheet1.zip › ╘¡╩╝╩2╛▌╔╧┤1⁄2/3 Metagenomics/C Species Alpha diversity boxplot/Species Alpha diversity boxplot.pdf]

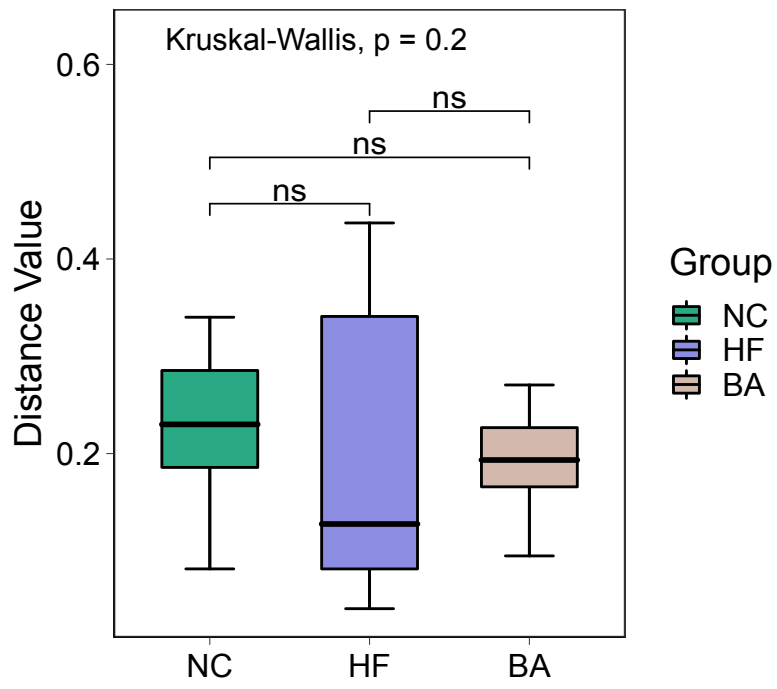

Supplement: Supplementary file 1 [file DataSheet1.zip › ╘¡╩╝╩2╛▌╔╧┤1⁄2/3 Metagenomics/D Species Beta diversity boxplot/Species Beta diversity boxplot_202504021957.pdf]

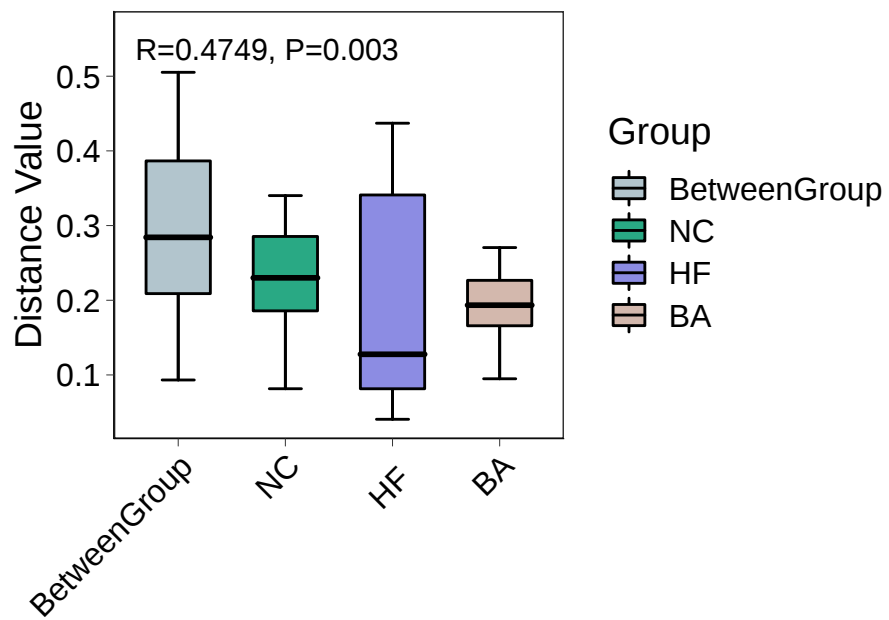

Supplement: Supplementary file 1 [file DataSheet1.zip › ╘¡╩╝╩2╛▌╔╧┤1⁄2/3 Metagenomics/E Species ANOSIM analysis/Species ANOSIM analysis_202504022005.pdf]

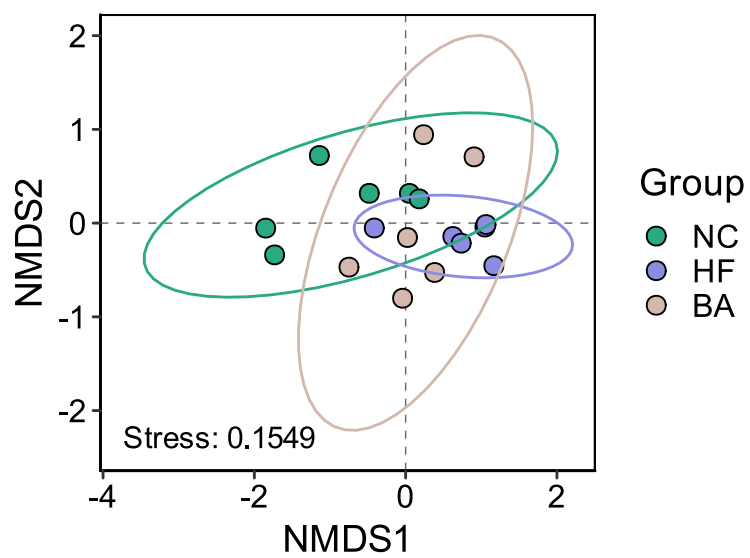

Supplement: Supplementary file 1 [file DataSheet1.zip › ╘¡╩╝╩2╛▌╔╧┤1⁄2/3 Metagenomics/F Species NMDS analysis/Species NMDS analysis_202504022009.pdf]

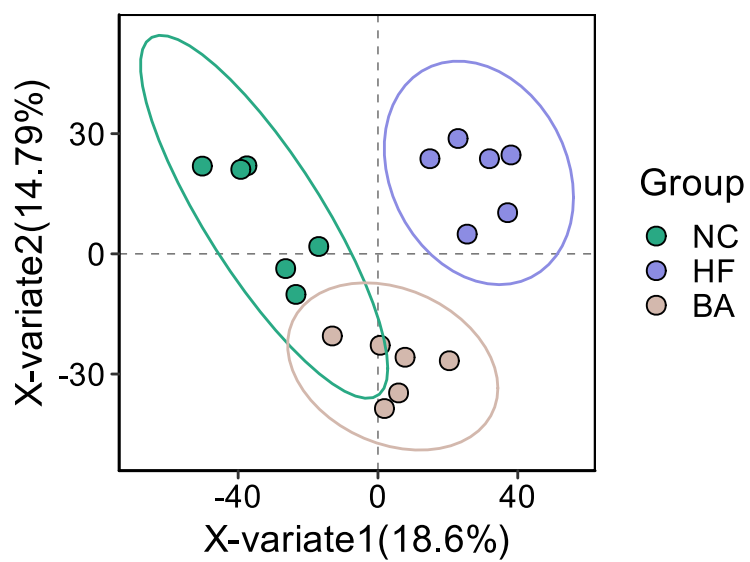

Supplement: Supplementary file 1 [file DataSheet1.zip › ╘¡╩╝╩2╛▌╔╧┤1⁄2/3 Metagenomics/G Functional PLS-DA analysis/Functional PLS-DA analysis_202504022012.pdf]

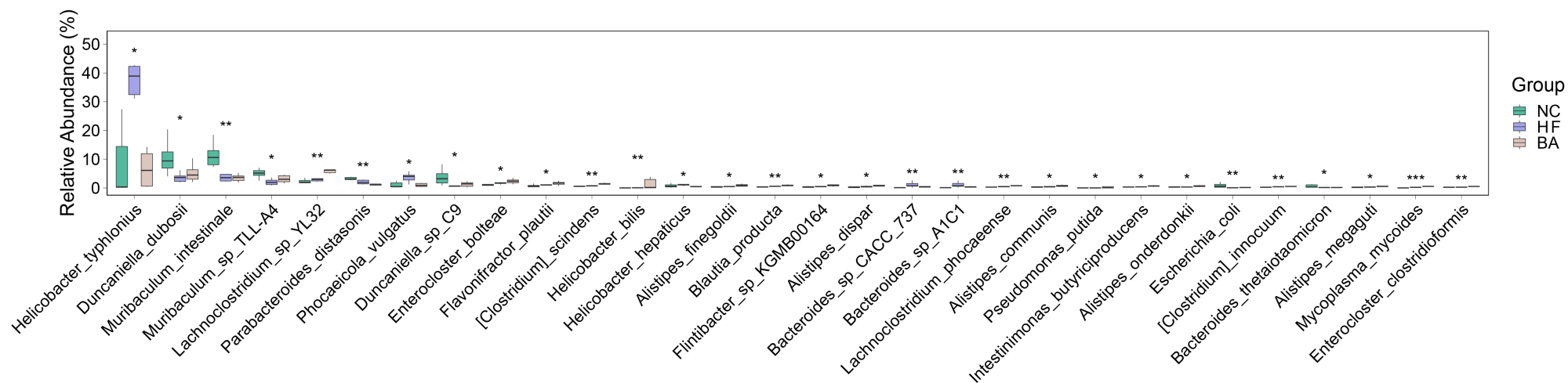

Supplement: Supplementary file 1 [file DataSheet1.zip › ╘¡╩╝╩2╛▌╔╧┤1⁄2/3 Metagenomics/H Differential species boxplot/Differential species boxplot.pdf]

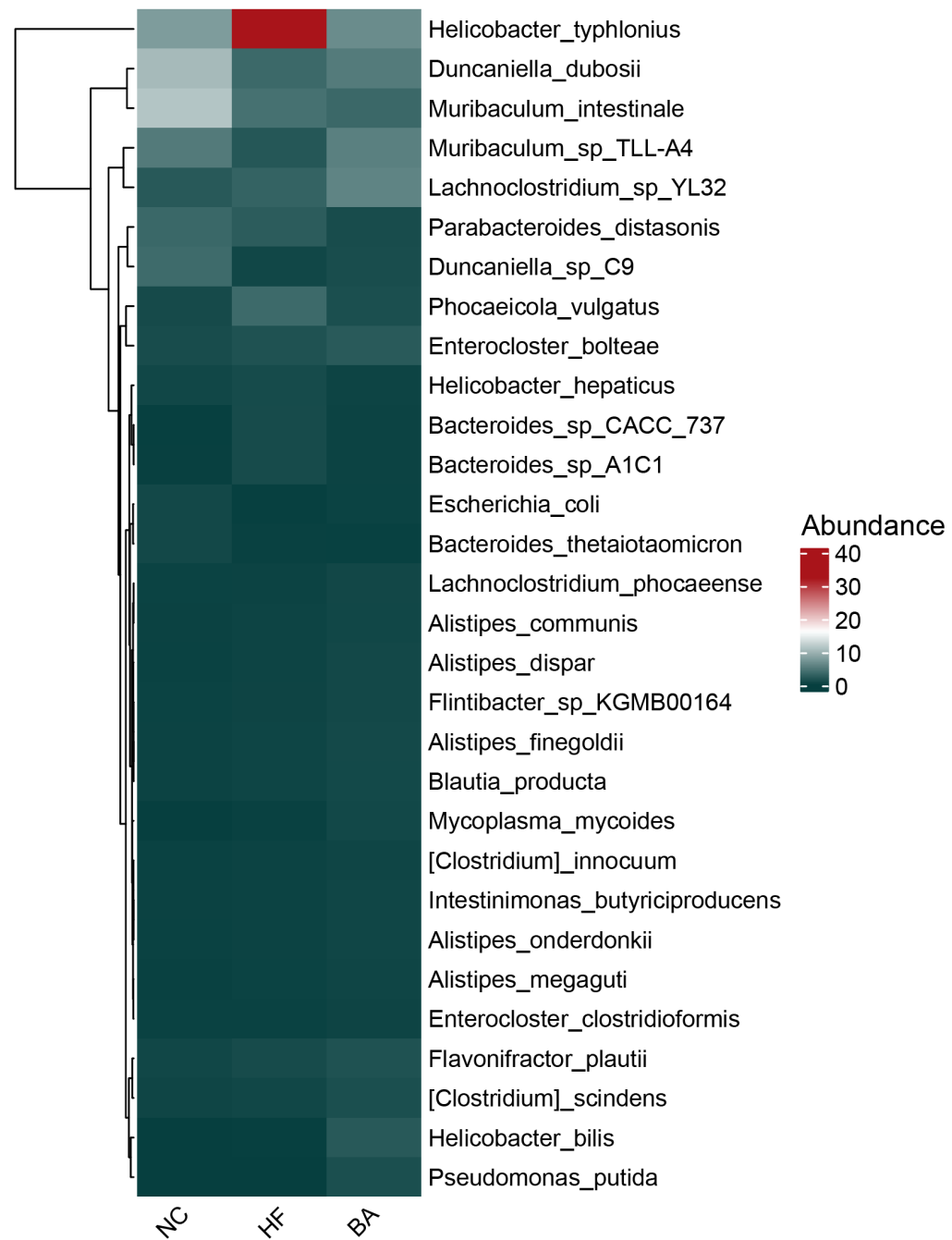

Supplement: Supplementary file 1 [file DataSheet1.zip › ╘¡╩╝╩2╛▌╔╧┤1⁄2/3 Metagenomics/I Species difference abundance heatmap/Species difference abundance heatmap.pdf]

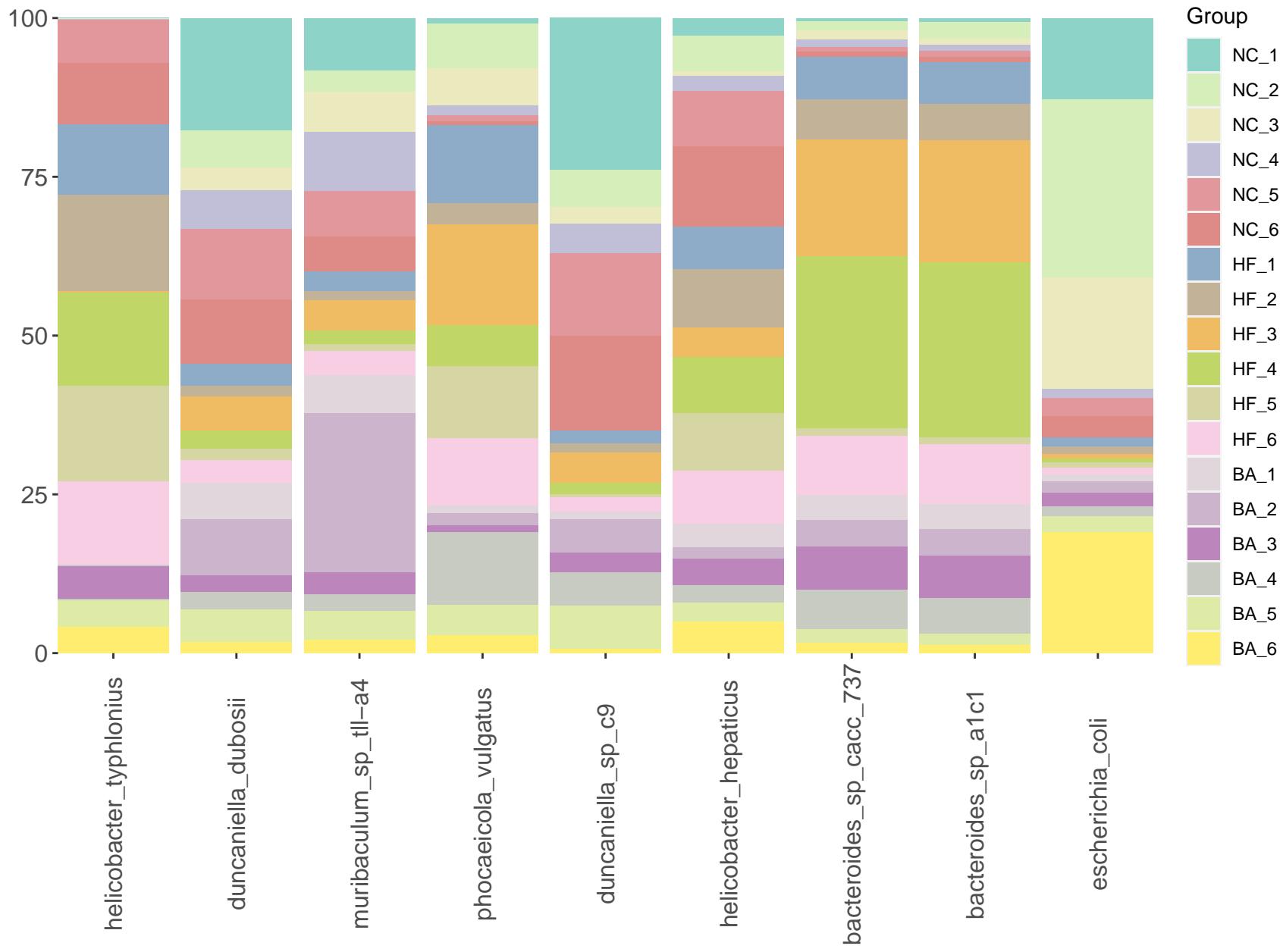

Supplement: Supplementary file 1 [file DataSheet1.zip › ╘¡╩╝╩2╛▌╔╧┤1⁄2/3 Metagenomics/J 9 species significantly affected by baicalin/Barplot.pdf]

火山图

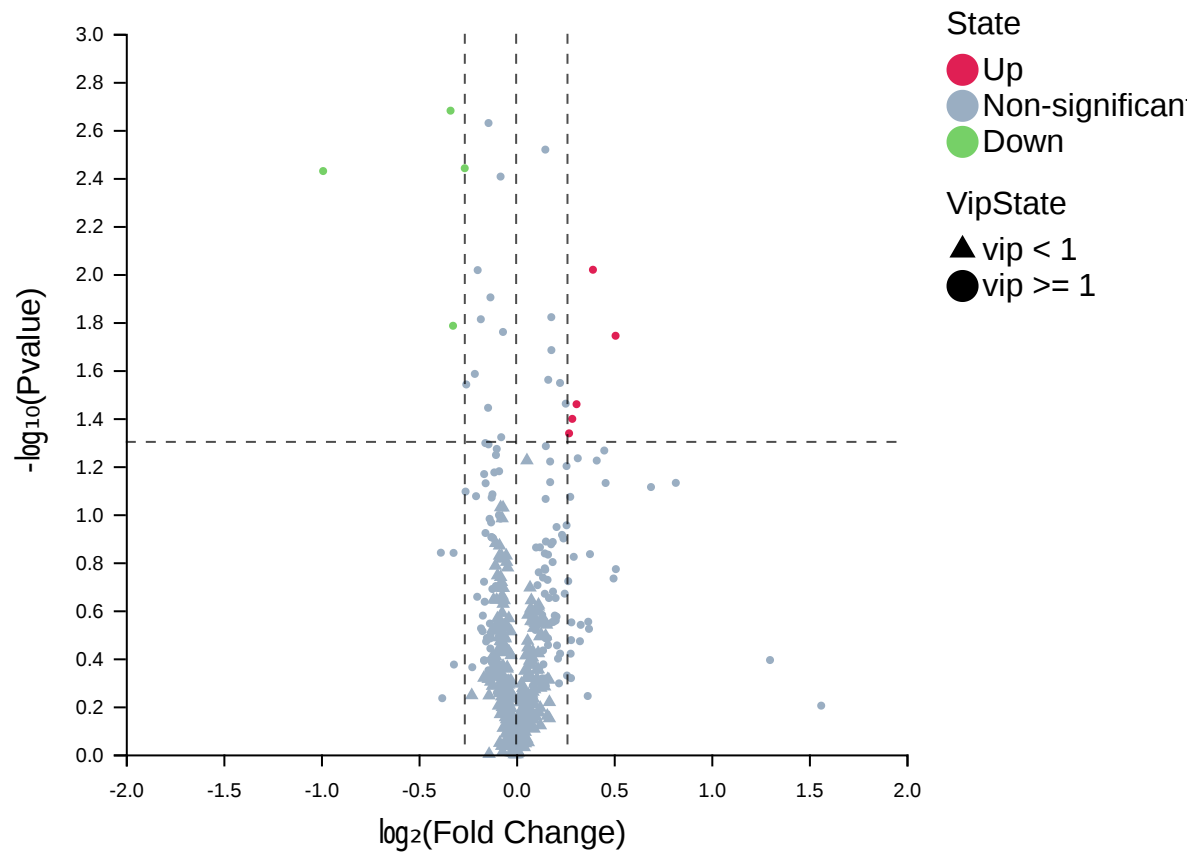

Supplement: Supplementary file 1 [file DataSheet1.zip › ╘¡╩╝╩2╛▌╔╧┤1⁄2/4 Lipidomics/Hippocampus/A VolcanoPlot/BAvsHF.pdf]

火山图

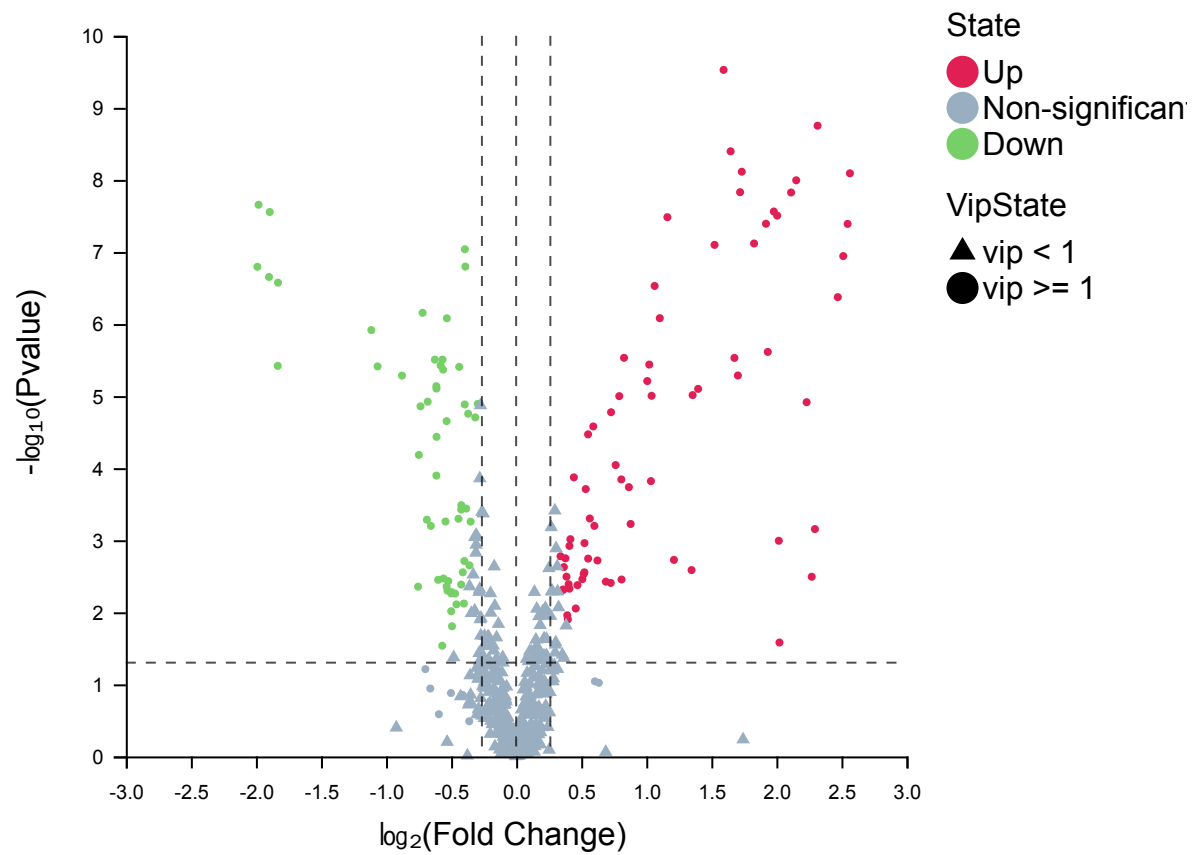

Supplement: Supplementary file 1 [file DataSheet1.zip › ╘¡╩╝╩2╛▌╔╧┤1⁄2/4 Lipidomics/Hippocampus/A VolcanoPlot/HFvsNC.pdf]

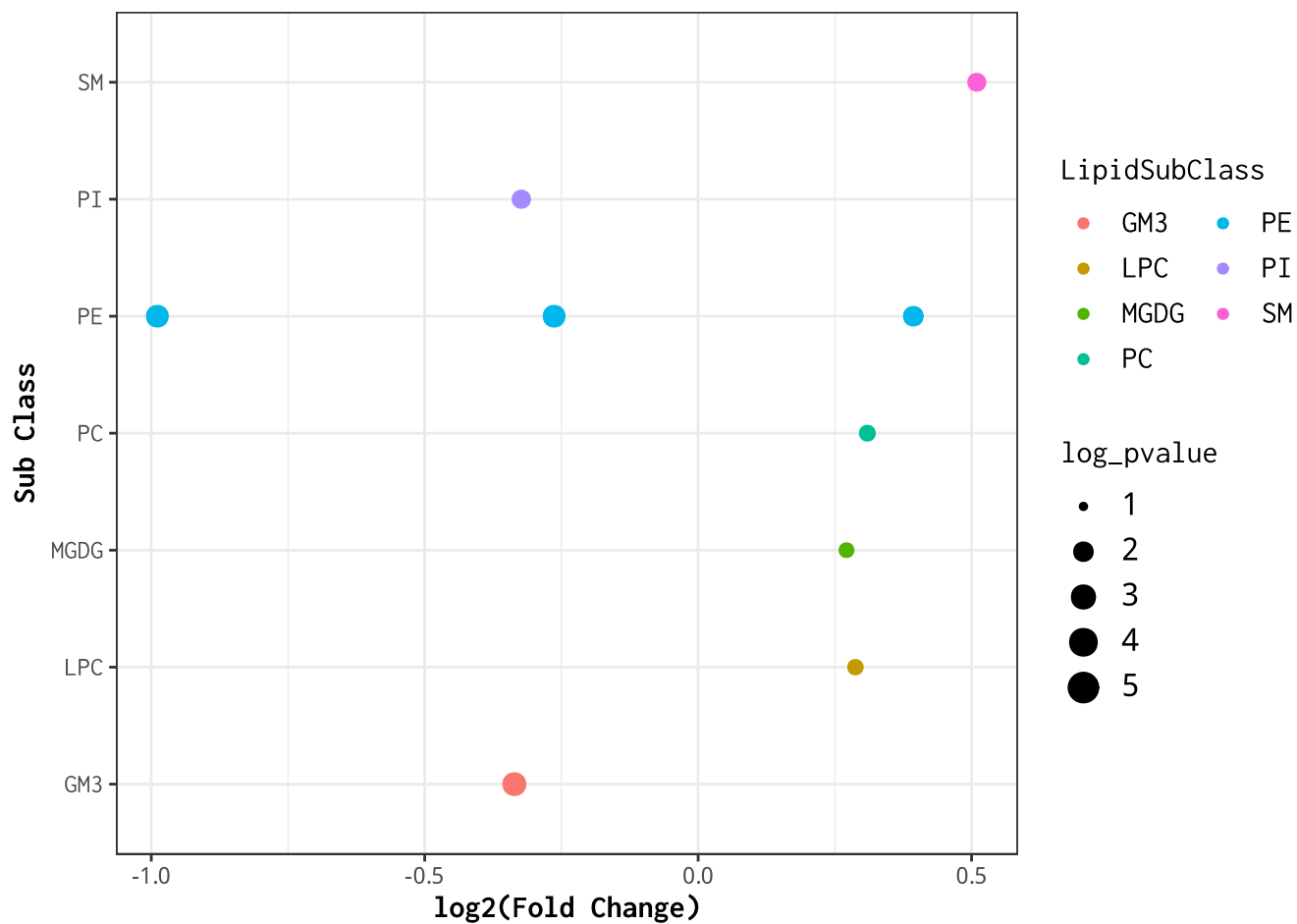

Supplement: Supplementary file 1 [file DataSheet1.zip › ╘¡╩╝╩2╛▌╔╧┤1⁄2/4 Lipidomics/Hippocampus/B Content analysis-bubble diagram/BAHvsHFH/Content analysis bubble diagram.pdf]

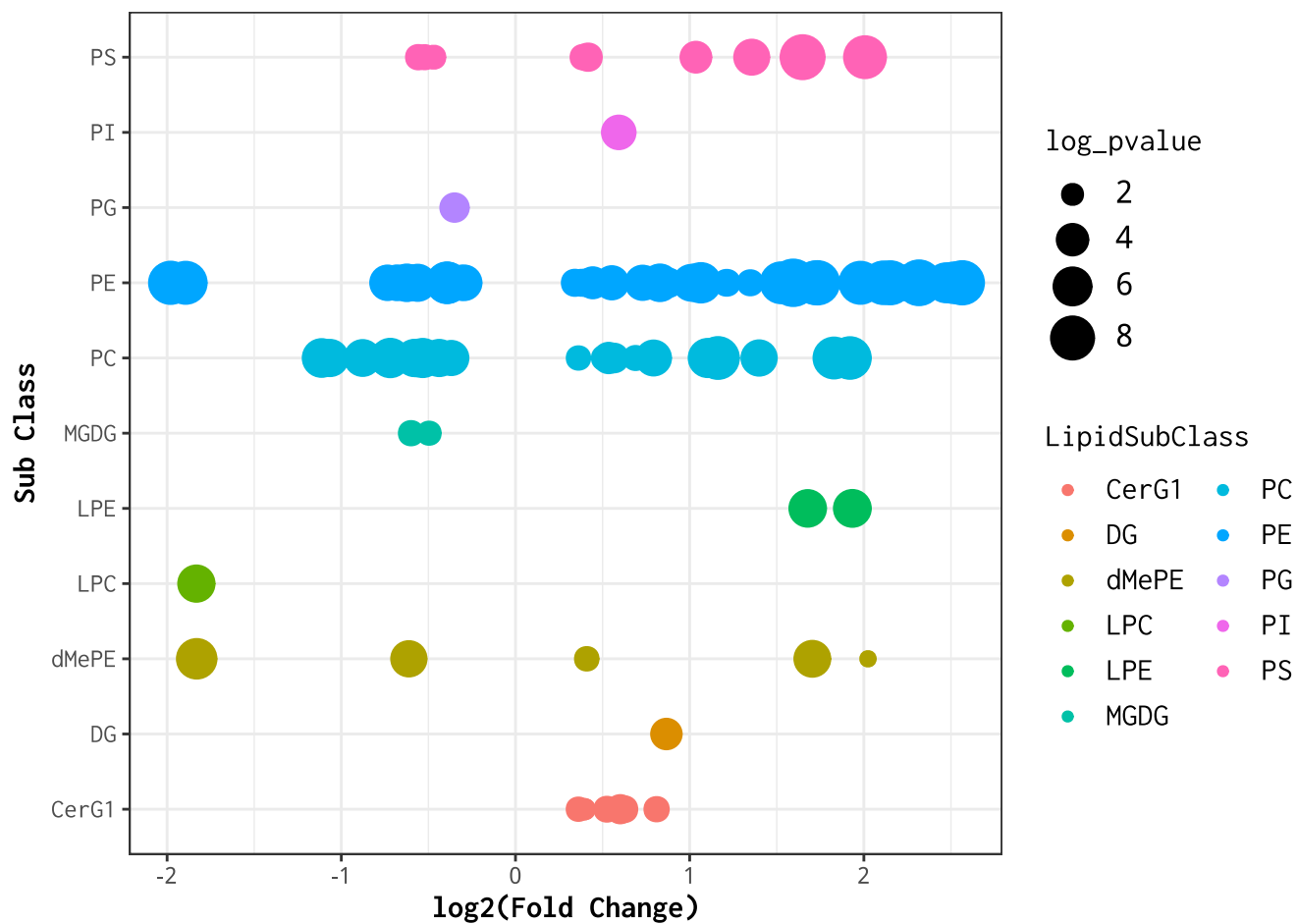

Supplement: Supplementary file 1 [file DataSheet1.zip › ╘¡╩╝╩2╛▌╔╧┤1⁄2/4 Lipidomics/Hippocampus/B Content analysis-bubble diagram/HFHvsNCH/Content analysis bubble diagram.pdf]

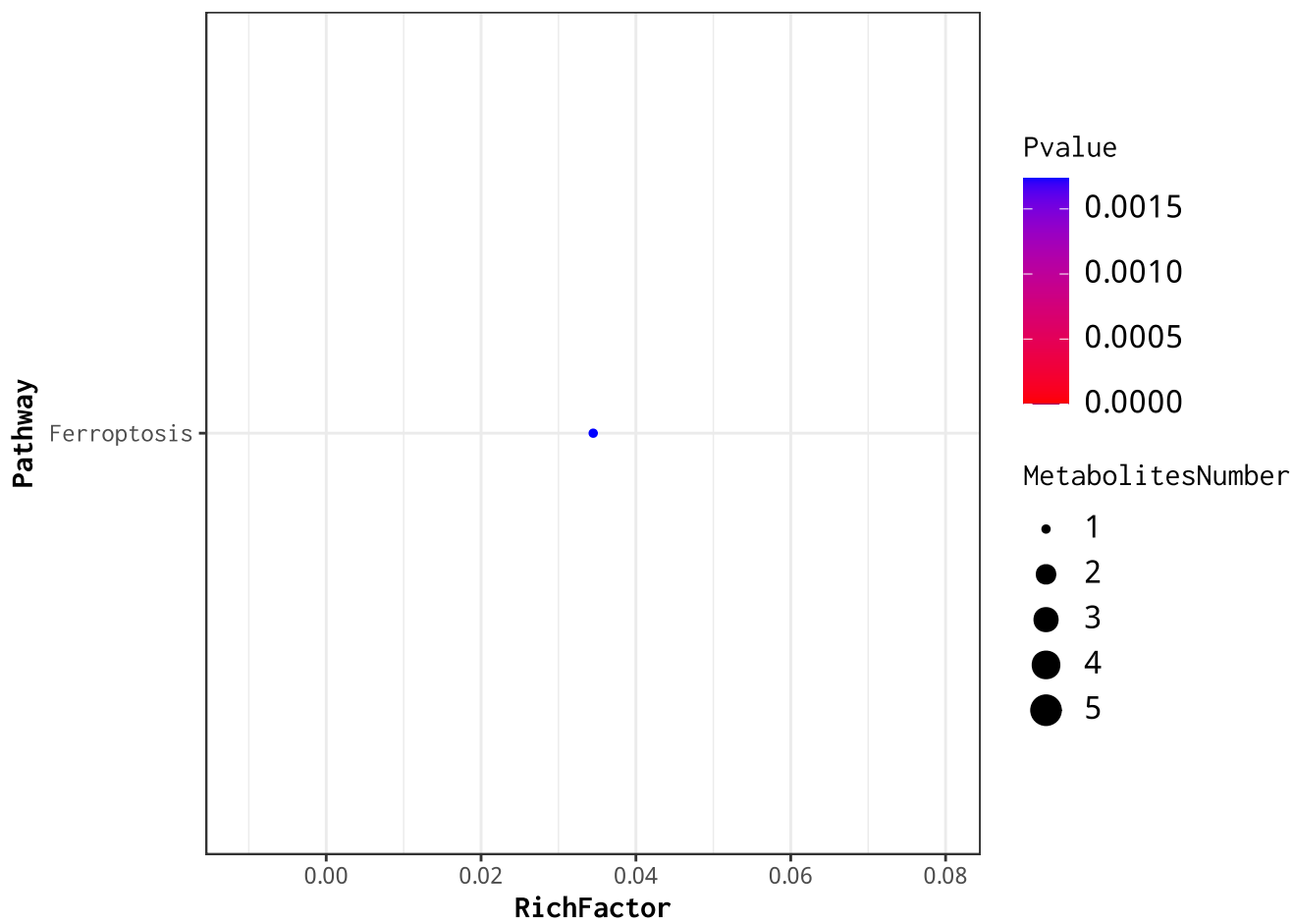

Supplement: Supplementary file 1 [file DataSheet1.zip › ╘¡╩╝╩2╛▌╔╧┤1⁄2/4 Lipidomics/Hippocampus/D Enrichment analysis-bubble diagram/Enrichment analysis-bubble diagram_202504031008.pdf]

火山图

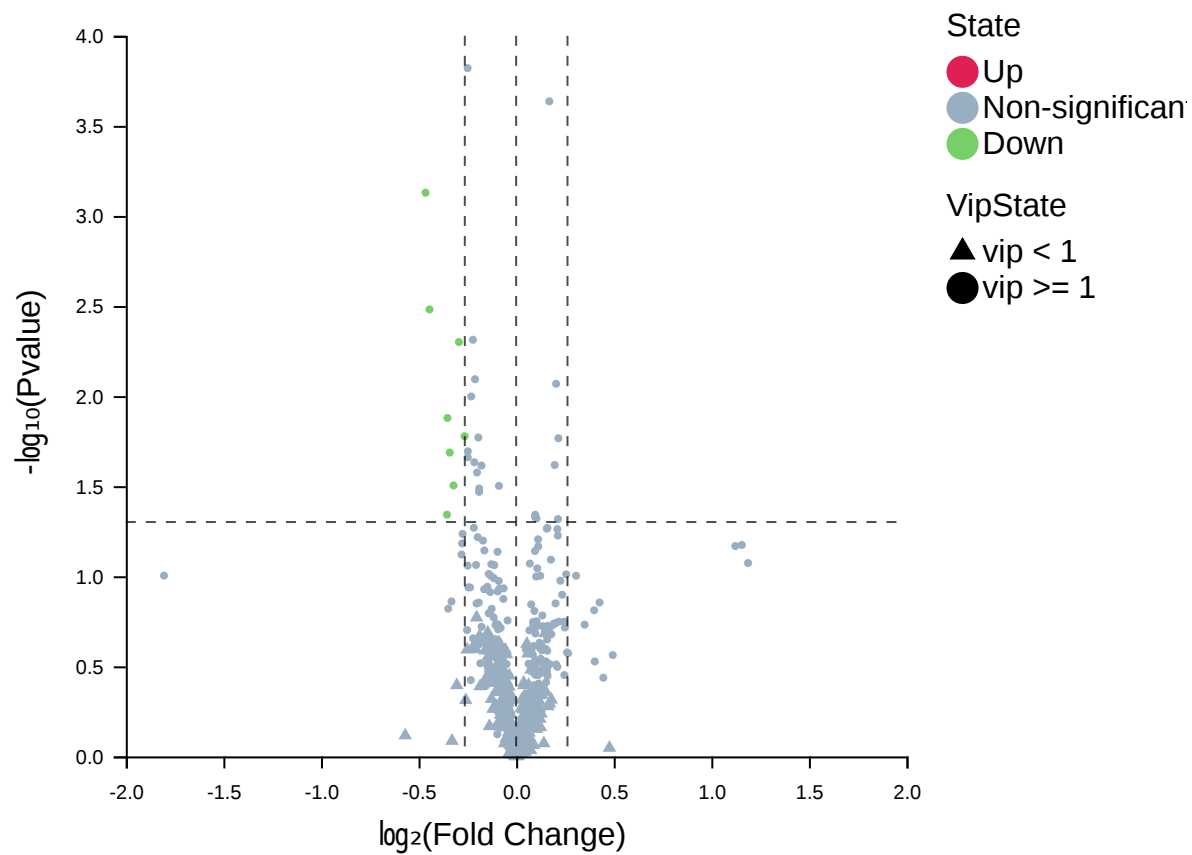

Supplement: Supplementary file 1 [file DataSheet1.zip › ╘¡╩╝╩2╛▌╔╧┤1⁄2/4 Lipidomics/Prefrontal cortex/A VolcanoPlot/BAPvsHFH.pdf]

火山图

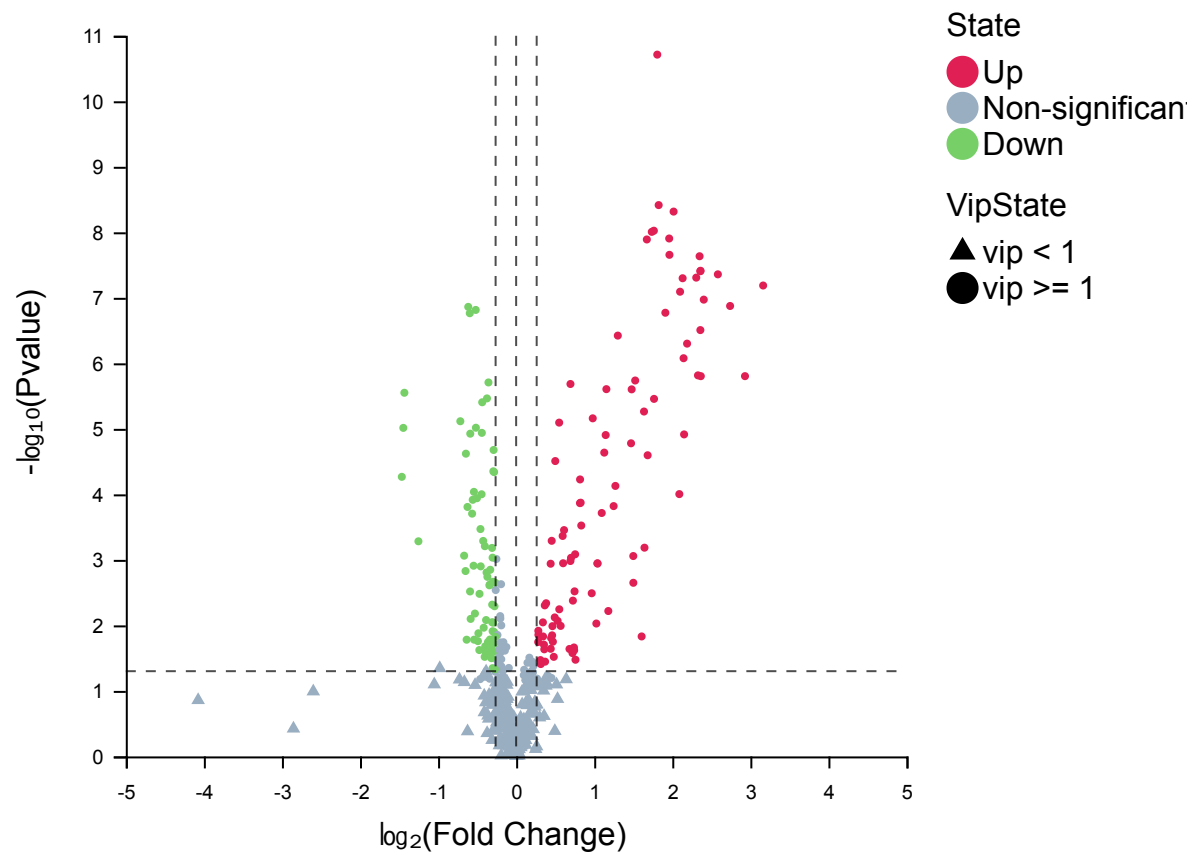

Supplement: Supplementary file 1 [file DataSheet1.zip › ╘¡╩╝╩2╛▌╔╧┤1⁄2/4 Lipidomics/Prefrontal cortex/A VolcanoPlot/HFHvsNCH.pdf]

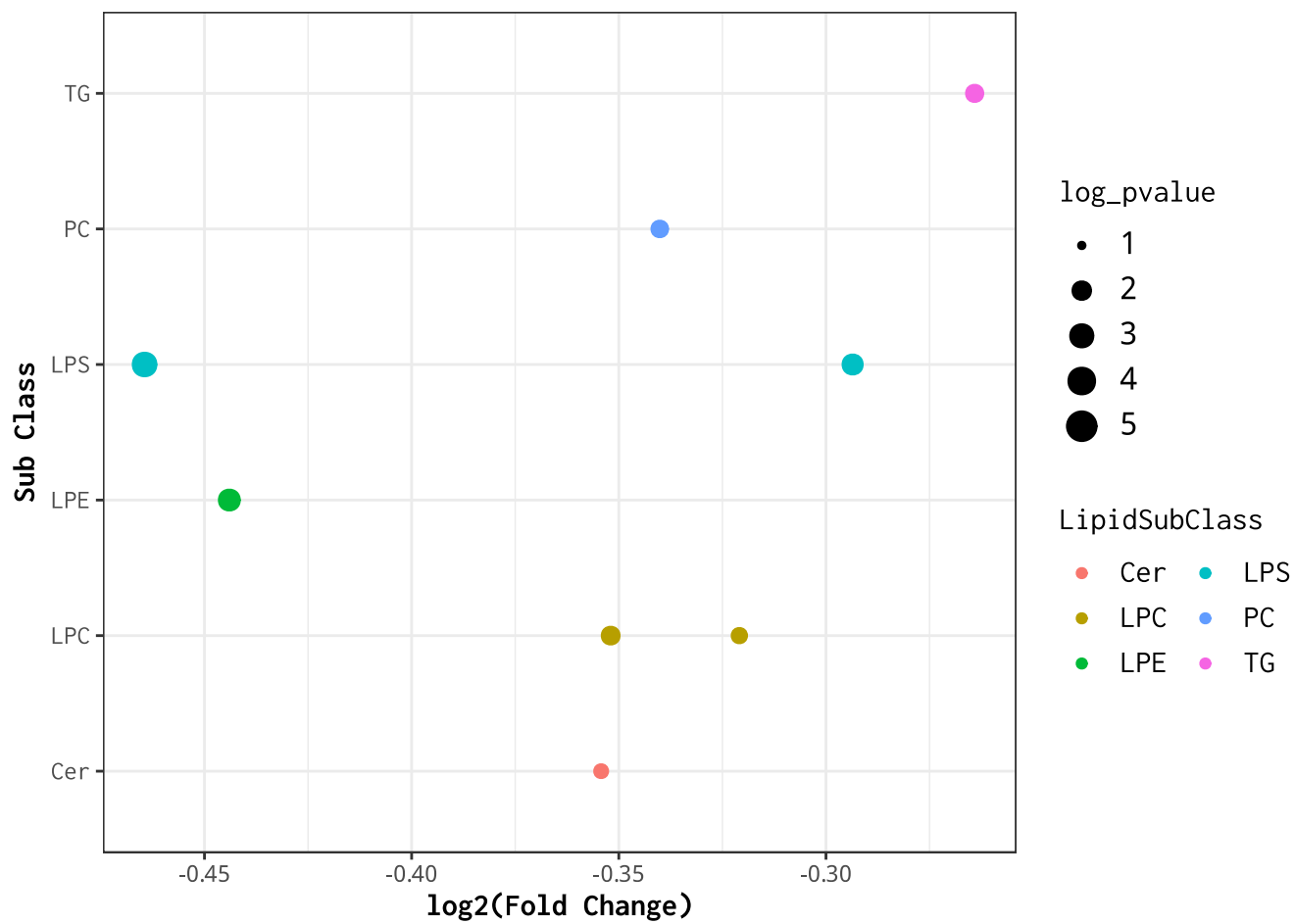

Supplement: Supplementary file 1 [file DataSheet1.zip › ╘¡╩╝╩2╛▌╔╧┤1⁄2/4 Lipidomics/Prefrontal cortex/B Content analysis-bubble diagram/BAPvsHFP/Content analysis bubble diagram.pdf]

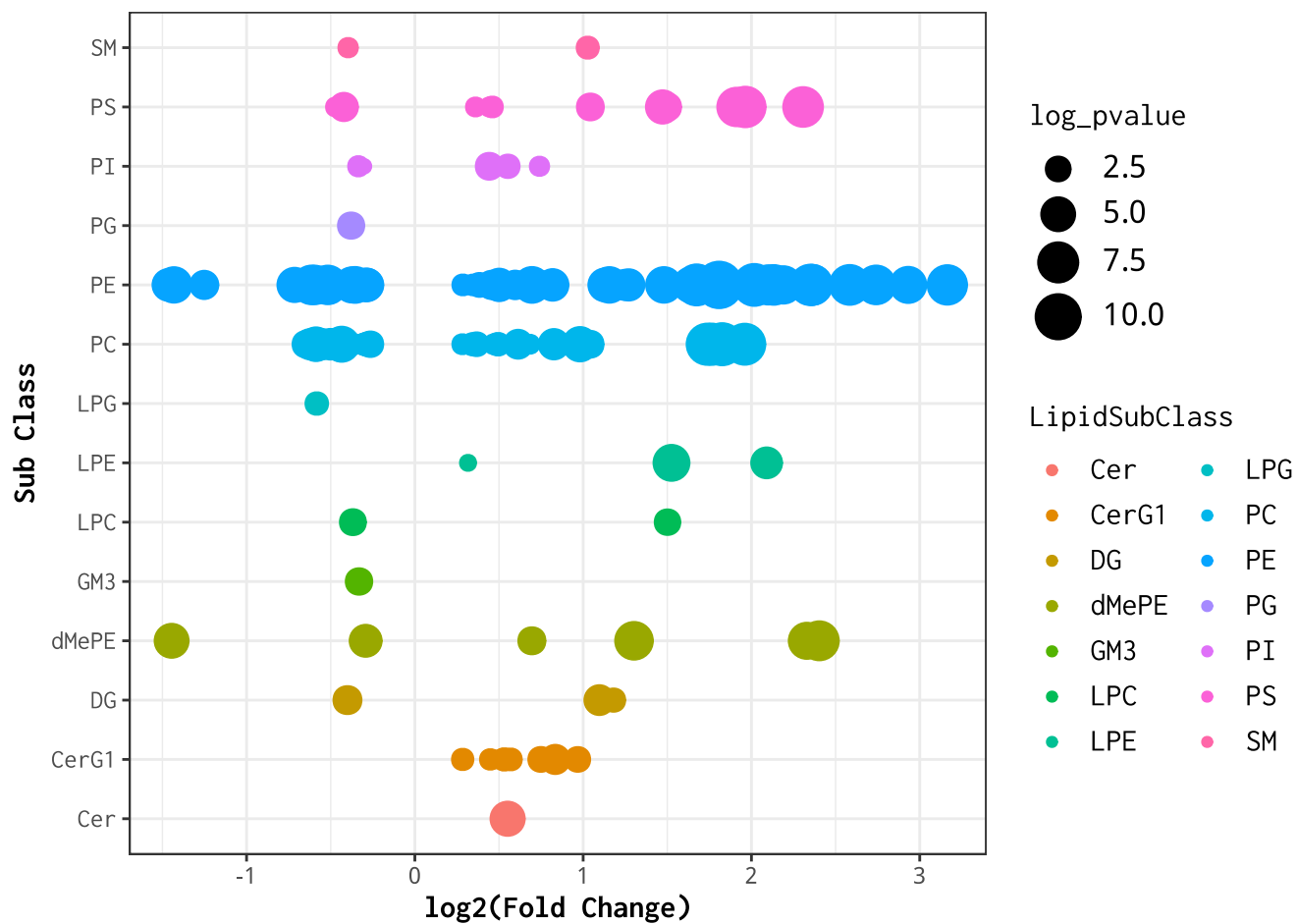

Supplement: Supplementary file 1 [file DataSheet1.zip › ╘¡╩╝╩2╛▌╔╧┤1⁄2/4 Lipidomics/Prefrontal cortex/B Content analysis-bubble diagram/HFPvsNCP/Content analysis bubble diagram.pdf]

**Pathway**

Ferroptosis

0.00

0.02

0.04

0.06

0.08

**RichFactor**

**Pvalue**

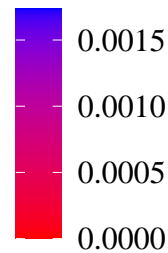

**MetabolitesNumber**

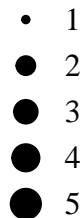

Supplement: Supplementary file 1 [file DataSheet1.zip › ╘¡╩╝╩2╛▌╔╧┤1⁄2/4 Lipidomics/Prefrontal cortex/D Enrichment analysis-bubble diagram/BubbleDiagram.pdf]

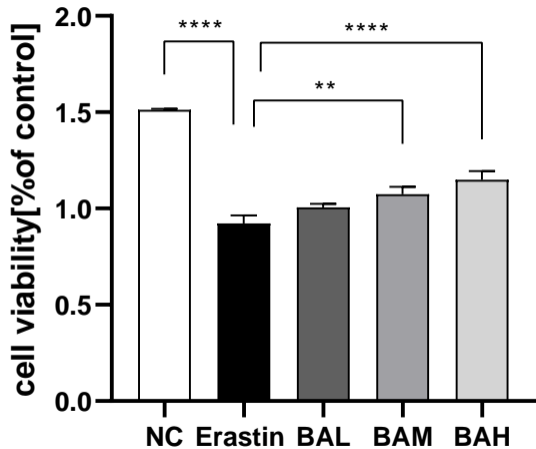

Supplement: Supplementary file 1 [file DataSheet1.zip › ╘¡╩╝╩2╛▌╔╧┤1⁄2/6 Cell experiments/A cck8/CCK8.pdf]

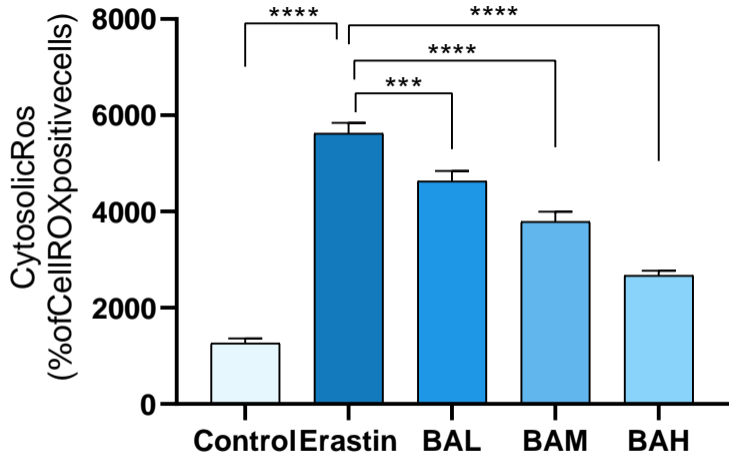

Supplement: Supplementary file 1 [file DataSheet1.zip › ╘¡╩╝╩2╛▌╔╧┤1⁄2/6 Cell experiments/B ROS/ROS(2).pdf]

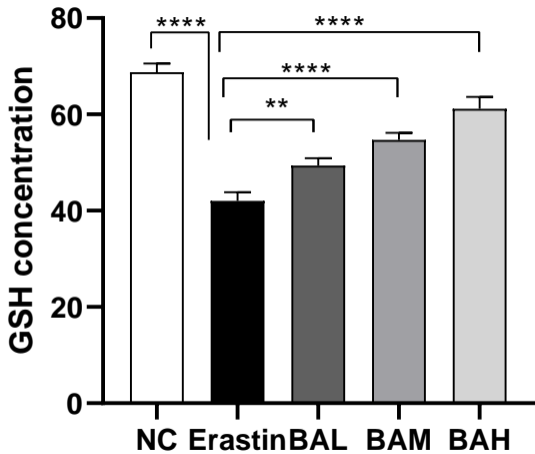

Supplement: Supplementary file 1 [file DataSheet1.zip › ╘¡╩╝╩2╛▌╔╧┤1⁄2/6 Cell experiments/C GSH/GSH.pdf]

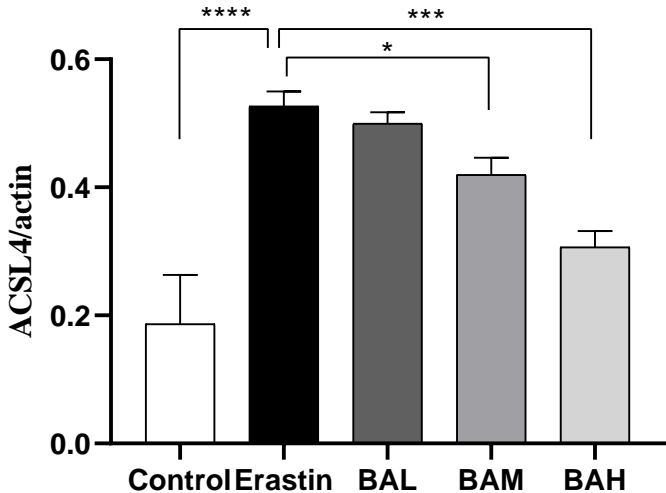

Supplement: Supplementary file 1 [file DataSheet1.zip › ╘¡╩╝╩2╛▌╔╧┤1⁄2/6 Cell experiments/DEF WB/ACSL4.pdf]

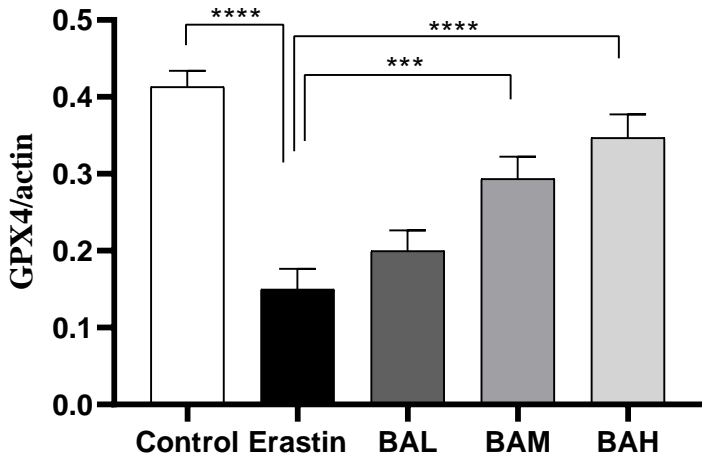

Supplement: Supplementary file 1 [file DataSheet1.zip › ╘¡╩╝╩2╛▌╔╧┤1⁄2/6 Cell experiments/DEF WB/GPX4.pdf]

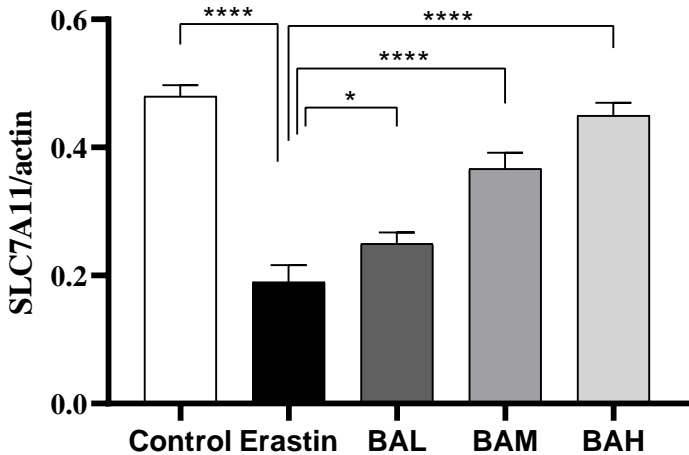

Supplement: Supplementary file 1 [file DataSheet1.zip › ╘¡╩╝╩2╛▌╔╧┤1⁄2/6 Cell experiments/DEF WB/SLCA11.pdf]
